# Profiles and Findings of Population-Based Esophageal Cancer Screening With Endoscopy in China: Systematic Review and Meta-analysis

**DOI:** 10.2196/45360

**Published:** 2023-06-01

**Authors:** He Li, Yi Teng, Xinxin Yan, Maomao Cao, Fan Yang, Siyi He, Shaoli Zhang, Qianru Li, Changfa Xia, Kai Li, Wanqing Chen

**Affiliations:** 1 Office of Cancer Screening, National Cancer Center of China/Cancer Hospital Chinese Academy of Medical Sciences and Peking Union Medical College Beijing China; 2 Department of Surgical Oncology and General Surgery Key Laboratory of Precision Diagnosis and Treatment of Gastrointestinal Tumors Ministry of Education, The First Affiliated Hospital of China Medical University Shenyang China

**Keywords:** esophageal cancer, screening, high-risk individuals, detection rates, China

## Abstract

**Background:**

Population-based esophageal cancer (EC) screening trials and programs have been conducted in China for decades; however, screening strategies have been adopted in different regions and screening profiles are unclear.

**Objective:**

We performed a meta-analysis to profile EC screening in China by positivity rate, compliance rate, and endoscopy findings, aiming to provide explicit evidence and recommendations for EC screening programs.

**Methods:**

English (PubMed, Embase) and Chinese (China National Knowledge Infrastructure, Wanfang) language databases were systematically searched for population-based EC screening studies in the Chinese population until December 31, 2022. A meta-analysis was performed by standard methodology using a random-effects model. Pooled prevalence rates were calculated for three groups: high-risk areas with a universal endoscopy strategy, rural China with a risk-stratified endoscopic screening (RSES) strategy, and urban China with an RSES strategy. Positive cases included lesions of severe dysplasia, carcinoma in situ, intramucosal carcinoma, submucosal carcinoma, and invasive carcinoma.

**Results:**

The pooled positivity rate of the high-risk population was higher in rural China (44.12%) than in urban China (23.11%). The compliance rate of endoscopic examinations was the highest in rural China (52.40%), followed by high-risk areas (50.11%), and was the lowest in urban China (23.67%). The pooled detection rate of positive cases decreased from 1.03% (95% CI 0.82%-1.30%) in high-risk areas to 0.48% (95% CI 0.25%-0.93%) in rural China and 0.12% (95% CI 0.07%-0.21%) in urban China. The pooled detection rate of low-grade intraepithelial neoplasia (LGIN) was also in the same order, being the highest in high-risk areas (3.99%, 95% CI 2.78%-5.69%), followed by rural China (2.55%, 95% CI 1.03%-6.19%) and urban China (0.34%, 95% CI 0.14%-0.81%). Higher detection rates of positive cases and LGIN were observed among males than among females and at older ages. The pooled early detection rate was 81.90% (95% CI 75.58%-86.88%), which was similar to the rates in high-risk areas (82.09%), in rural China (80.76%), and in urban China (80.08%).

**Conclusions:**

Under the current screening framework, a higher screening benefit was observed in high-risk areas than in other regions. To promote EC screening and reduce the current inequality of screening in China, more focus should be given to optimizing strategies of high-risk individual assessment and surveillance management to improve compliance with endoscopic examination.

**Trial Registration:**

PROSPERO CRD42022375720; https://www.crd.york.ac.uk/prospero/display_record.php?RecordID=375720

## Introduction

Esophageal cancer (EC) represents a global public health burden, with the primary pathological type being esophageal squamous cell carcinoma (ESCC) [1]. More than 50% of global ESCC cases occur in China, causing severe disease and an economic burden for the country [2,3]. A series of prevention and control strategies for EC have been implemented in China since the 1960s, especially in well-known high-risk areas such as Linzhou of Henan Province, where the incidence and mortality of EC were estimated to be approximately five times higher than the national average levels [4]. These strategies and actions have brought success, contributing to an average 4.5% reduction in the incidence and mortality of EC in China since 2000 [5]. In addition, the results of a screening program in high-risk areas in China demonstrated the effectiveness of EC screening with endoscopy examination in reducing EC incidence and mortality [6], which provided high-quality evidence and strong application recommendations for international EC screening and early detection and treatment.

Three organized screening programs have been launched based on the National Key Public Health Project since 2005 to conduct population-based EC screening in the high-risk areas of Huaihe River and urban China [7]. The primary aim of these programs is to reduce the incidence and mortality of EC and to explore a suitable and feasible EC screening strategy. In practice, two main EC screening strategies have been adopted and implemented in China. One is the universal endoscopy screening strategy, which is used in high-risk areas. The other is the risk-stratified endoscopic screening (RSES) strategy, which provides endoscopies for a limited group of individuals at high risk of EC and is currently widely implemented in nonhigh-risk areas [7]. These programs and other population-based ESCC screening studies have covered more than 150 counties or cities in China [7], providing and accumulating a great deal of experience and real-world data for ESCC prevention and control in China and internationally.

Clear and comprehensive knowledge of the profile of EC screening, including the positivity rate of high-risk individuals for EC, compliance with endoscopy screening, and endoscopy findings in real-world EC screening programs, has potential public health value. For instance, such knowledge would help health policy makers understand the actual acceptability of EC screening, direct benefits from EC screening, and burden of surveillance endoscopy. In addition, this crucial real-world evidence would promote the further implementation of EC screening programs in China and other countries that face a high EC burden. To the best of our knowledge, EC screening profiles in China are unclear based on existing studies and the literature. To obtain a comprehensive profile of EC screening in China, we performed this systematic review and meta-analysis to estimate the positivity rate of EC among high-risk individuals, compliance with endoscopy screening, and endoscopy findings by high-risk and nonhigh-risk areas (rural China and urban China). By summarizing these indices, we hope to provide more explicit evidence and recommendations for Chinese EC prevention and control and to promote further EC screening in other countries facing the threat of a high EC burden, which has great importance for public health.

## Methods

### Design

The protocol for this systematic review was published in the International Prospective Register of Systematic Reviews (PROSPERO; registration number CRD42022375720) and the review was designed following the PRISMA (Preferred Reporting Items for Systematic Reviews and Meta-Analyses) guidelines 2020 [8].

### Data Sources and Search Strategy

The English-language (PubMed, Embase) and Chinese (China National Knowledge Infrastructure, Wanfang) databases were systematically searched for population-based EC screening studies in the Chinese population until December 31, 2022, in any language. The following relevant Medical Subject Heading (MeSH) terms and key words were used for the search: esophageal neoplasms, screening, and China (see Table S1 in Multimedia Appendix 1 for the full search strategy). Two researchers (HL, YT) performed the literature search independently and discrepancies were resolved by consultation with a third researcher (XY).

### Study Selection

#### Inclusion Criteria

Studies were selected based on the following inclusion criteria: (1) studies that reported compliance with endoscopy examination and/or endoscopy findings from population-based EC screening in the Chinese population without restrictions on age; and (2) cross-sectional studies, cohort studies, and randomized controlled trials that reported baseline findings.

#### Exclusion Criteria

Studies were excluded if they (1) were nonpopulation-based screening studies, (2) lacked information on compliance rates with endoscopy exam and endoscopy findings of positive cases or low-grade intraepithelial neoplasia (LGIN), and (3) were studies with duplicated data for all outcomes of interest published elsewhere or parts of another study from the same geographical location.

### Data Extraction and Quality Assessment

For each included study, two reviewers (HL, YT) independently assessed the studies and extracted the data for analysis. The extracted information included the first author and year of publication, study periods, study region, number of study centers, screening strategy (universal endoscopy screening/RSES), sex (proportion of males), age, available data of sex-specific and age-specific subgroups, and available data of early detection among patients with positive screening results.

The study quality and risk of bias were assessed using an instrument developed by Hoy and colleagues [9] for population-based prevalence studies. The tool has 10 questions, and a score of 1 (yes) or 0 (no) was assigned for each item (see Table S2 in Multimedia Appendix 1). Scores were summed across items to generate an overall quality score that ranged from 0 to 10, and studies were then classified as having a low (scores≥8), moderate (scores of 6-7), or high (scores≤5) risk of bias. This method has been used in previous systematic reviews. This assessment tool was tested in previous studies; it is easy to use and deals with risk of bias well.

### Outcomes Assessed

The pooled outcomes assessed included the positivity rate of high-risk individuals for EC/upper gastrointestinal (UGI) cancer, compliance rates to endoscopy examinations, prevalence of endoscopy findings (positive cases, LGIN, and negative endoscopy), and early detection rate. The definition of each index is described in Table S3 of Multimedia Appendix 1. Positive cases included lesions of severe dysplasia, carcinoma in situ, intramucosal carcinoma, submucosal carcinoma, and invasive carcinoma. LGIN included lesions of mild dysplasia and moderate dysplasia. Negative endoscopy findings were defined as a baseline endoscopic examination that did not reveal any of the dysplasia lesions mentioned above.

### Statistical Analysis

We used meta-analysis techniques with logit transformation to calculate the pooled proportions along with the corresponding 95% CIs under a random-effects model. We calculated the pooled prevalence in three populations with different screening strategies: (1) high-risk areas with a universal endoscopy strategy, (2) rural China with an RSES strategy, and (3) urban China with an RSES strategy. Analyses of the pooled prevalence of endoscopy findings by sex and age group (40-49, 50-59, and 60-69 years) were also performed. We performed sensitivity analyses by publication year, sample size, and the number of study centers to evaluate the robustness of the study.

Heterogeneity between studies was assessed with the *I*² statistic, which estimates the percentage of the total variation across studies due to true between-study differences. Generally, *I²*values greater than 60%-70% indicate the presence of substantial heterogeneity. Publication bias was inspected visually on a funnel plot and by the Egger test; *P*<.05 was considered statistically significant. All meta-analyses were carried out using R software (version 4.1.0; R Foundation for Statistical Computing, Vienna, Austria).

### Ethical Considerations

This is a meta-analysis of published studies and data, and did not involve active human participants and/or animals. The present study used only publicly available summary-level statistics and did not involve individual information. Therefore, formal consent, informed consent, institutional review board approval, and ethics approval are not applicable and/or not needed.

## Results

### Search Results

**Figure 1:**
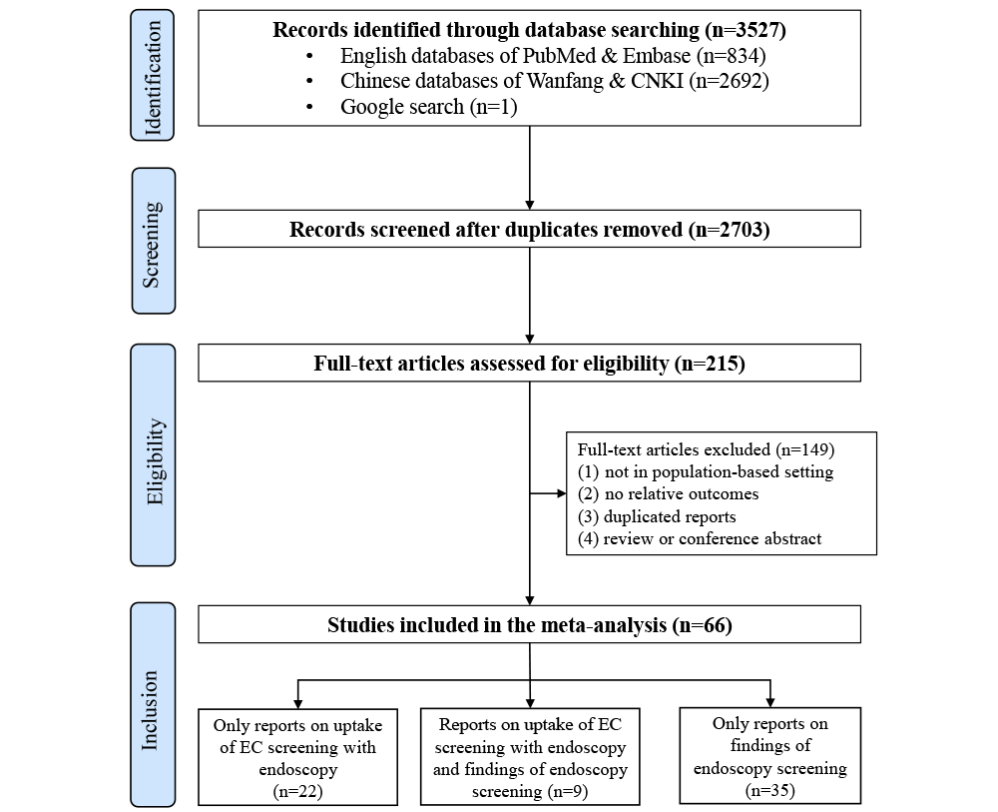
Flowchart presenting the selection of studies for inclusion in the systematic review and meta-analysis. CNKI: China National Knowledge Infrastructure; EC: esophageal cancer.

A schematic diagram of study selection is provided in Figure 1. The initial search identified 3527 records, and 2703 titles and abstracts were screened after removing duplicates. A total of 215 full-length articles were evaluated in detail and 66 studies were included in the final analysis. Among these studies, 22 studies only reported the compliance of EC screening with endoscopy, 35 studies only reported the findings of endoscopy examinations, and 9 studies reported both outcomes.

### Study Characteristics and Quality Assessment

The characteristics of the 66 included studies are outlined in Table S4 of Multimedia Appendix 1, and the summary characteristics about reporting compliance with endoscopy examinations and endoscopy findings are summarized in Table 1 and Table 2, respectively. Eligible ages for EC screening were 40-69 years in all studies conducted in high-risk areas and rural China, except for one study performed in Hua County with an age range of 25-65 years [10]. Eligibility ages in urban China were primarily 40-74 years, and the remaining studies recommended 40-69 years.

A total of 2,472,920 asymptomatic individuals were included in 31 studies that reported compliance with endoscopy examinations, one of which reported data from both rural and urban China [11]. Therefore, 2,006,235 individuals were from high-risk areas during 1999-2017 (n=12) [6,10,12-21], 125,473 individuals were from rural China during 2010-2017 (n=2) [11,22], and 341,212 individuals were from urban China during 2012-2019 (n=18) [11,23-39], as shown in Table 1 and Table S4 of Multimedia Appendix 1. The details of the high-risk assessment strategy to select targets to undergo further endoscopy screening are summarized in Table S5 of Multimedia Appendix 1.

A total of 997,004 individuals had reports of endoscopy findings in 44 studies, among which one study reported endoscopy findings in high-risk areas, rural China, and urban China [11]. Therefore, a total of 876,170 individuals aged 40-69 years (n=36 studies) [6,11,12,14-17,40-68] from high-risk areas underwent endoscopic examinations, where a universal endoscopy strategy was adopted. A total of 102,413 individuals with a high risk of EC (n=4 studies) [11,69-71] aged 40-69 years in rural China and 18,421 individuals with a high risk of EC/UGI cancer (n=6 studies) [11,29,33,72-74] aged 40-74 years in urban China underwent endoscopic examinations where the RSES strategy was adopted (Table 2). The number of individual study samples ranged from 1151 [74] to 116,630 [67] across all included studies. Study participants were enrolled from 1999 to 2019 in high-risk areas, followed by 2007 to 2017 in rural China and 2015 to 2019 in urban China. Study regions also presented disparities: study regions in high-risk areas mainly consisted of north China (22%) and southwest China (22%), followed by eastern China (19%), central China (17%), and northwest China (11%). Study regions in rural China were mostly concentrated in east China (50%) and central China (25%). Study regions in urban China were relatively evenly distributed across the country, except in central and northeast China (Table 2, Table S4 in Multimedia Appendix 1).

Of the 31 studies reporting compliance with EC screening using endoscopy, 12 (39%) and 19 (61%) were classified as having a low and moderate risk of bias, respectively (Table S6 of Multimedia Appendix 1). Of the 44 studies that reported findings of endoscopy examinations, 39 (89%) and 5 (11%) were classified as having a low and moderate risk of bias, respectively (Table S7 in Multimedia Appendix 1).

**Table 1:** Main characteristics of the 31 included studies that reported compliance with endoscopy exams in population-based esophageal cancer screening in China by region.

Characteristics		High-risk areas	Rural China	Urban China
Age range (years)		40-69^a^	40-69	40-74^b^
Screening strategy		Universal endoscopy screening	Risk-stratified endoscopy screening	Risk-stratified endoscopy screening
Initial screening		No	Risk assessment	Risk assessment
Examination technique		Endoscopy with pathology	Endoscopy with pathology	Endoscopy with pathology
Number of studies^c^		12	2	18
Year of enrollment		1999-2017	2010-2017	2012-2019
Year of publication		2003-2021	2020-2021	2016-2021
Number of participants		2,006,235	125,473	341,212
**Representativeness, n (%)**				
	Single center	10 (83.33)	0 (0.00)	12 (66.67)
	Multiple centers within one province	0 (0.00)	0 (0.00)	5 (27.78)
	Multiple centers across two provinces or more	2 (16.67)	2 (100.00)	1 (5.55)
**Regions, n (%)**				
	Central China	1 (8.33)	0 (0.00)	3 (16.67)
	East China	1 (8.33)	0 (0.00)	6 (33.32)
	North China	4 (33.33)	0 (0.00)	2 (11.11)
	South China	0 (0.00)	0 (0.00)	2 (11.11)
	Northeast China	0 (0.00)	0 (0.00)	1 (5.56)
	Southwest China	3 (25.00)	0 (0.00)	1 (5.56)
	Northwest China	1 (8.33)	0 (0.00)	2 (11.11)
	Multiple regions	2 (16.68)	2 (100.00)	1 (5.56)

^a^The age of eligibility of one study in high-risk areas was 25-65 years and that of the others was 40-69 years.

^b^The age of eligibility of one study in urban China was >35 years and that of the others was 40-69 years.

^c^One study reported compliance with endoscopy examinations in both rural China and urban China.

**Table 2:** Main characteristics of the 44 included studies that reported endoscopy findings in population-based esophageal cancer screening in China by region.

Characteristics		High-risk areas	Rural China	Urban China
Number of studies^a^		36	4	6
Year of enrollment		1999-2019	2007-2017	2015-2019
Year of publication		2003-2022	2019-2020	2017-2021
Number of participants		876,170	102,413	18,421
Age range (years)		40-69^b^	40-69	40-74
**Representativeness, n (%)**				
	Single center	20 (55.56)	0 (0.00)	3 (50.00)
	Multiple centers within one province	13 (36.11)	3 (75.00)	2 (33.33)
	Multiple centers across two provinces or above	3 (8.33)	1 (25.00)	1 (16.67)
**Regions, n (%)**				
	Central China	6 (16.67)	1 (25.00)	0 (0.00)
	East China	7 (19.44)	2 (50.00)	1 (16.67)
	North China	8 (22.22)	0 (0.00)	1 (16.67)
	South China	0 (0.00)	0 (0.00)	1 (16.67)
	Northeast China	0 (0.00)	0 (0.00)	0 (0.00)
	Southwest China	8 (22.22)	0 (0.00)	1 (16.67)
	Northwest China	4 (11.11)	0 (0.00)	1 (16.67)
	Multiple regions	3 (8.34)	1 (25.00)	1 (16.67)

^a^One study reported endoscopy findings in high-risk areas, rural China, and urban China.

^b^The age of eligibility of one study in high-risk areas was 25-65 years and that of the others was 40-69 years.

### Pooled Outcomes

#### Pooled Positivity Rates of the High-Risk Population and Compliance With Endoscopic Examinations

**Figure 2:**
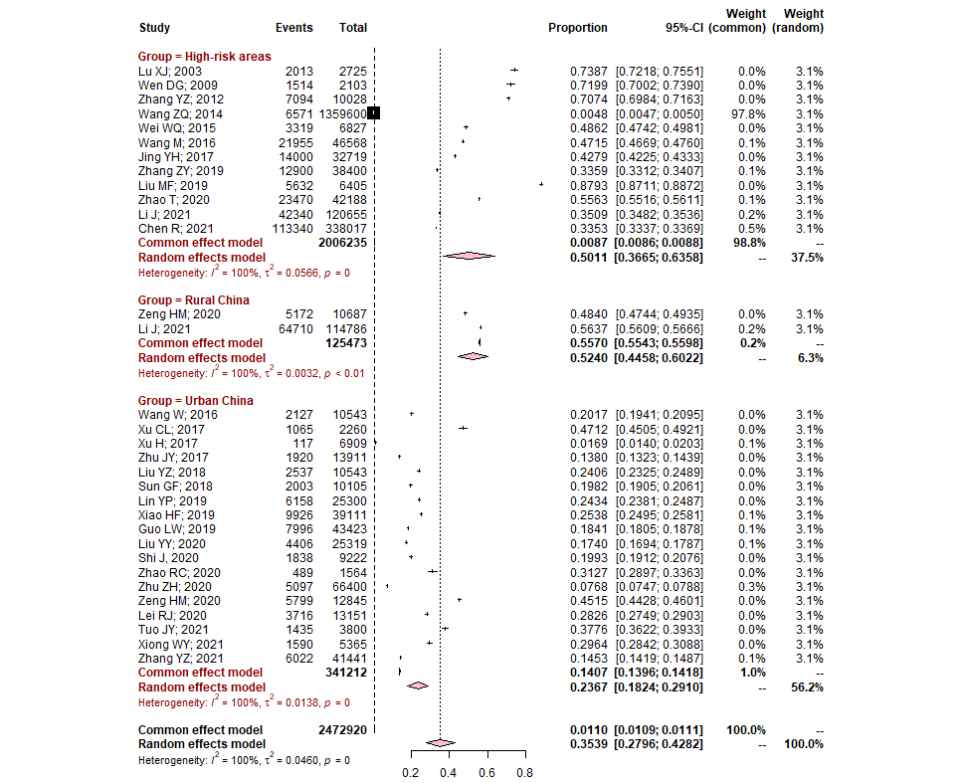
Pooled compliance rates of endoscopy examinations in population-based esophageal cancer screening by region (high-risk areas, rural China, and urban China).

All pooled results are summarized in Table 3. A total of 373,516 (in rural China) and 1,449,536 (in urban China) individuals of the asymptomatic population completed individualized risk assessment by professional staff from EC screening programs or trials. The pooled positivity rate of the high-risk population in rural China was higher than that in urban China; the details are shown in Figure S1 of Multimedia Appendix 1. The overall compliance rate with endoscopic examinations among the 31 included studies was 35.39% (95% CI 27.96%-42.82%), covering 2,472,920 eligible individuals for endoscopic examinations. The compliance rate was the highest in rural China, followed by high-risk areas, and was the lowest in urban China, as shown in Figure 2 and Table 3.

**Table 3:** Summary of pooled positivity rates of high-risk individuals, compliance rates of endoscopy examinations, and prevalence of endoscopy findings in population-based esophageal cancer (EC) screening in China by region.

Assessed metrics in population-based EC screening		High-risk areas		Rural China			Urban China	
		Pooled rate, % (95% CI)	*I* ^2^	Pooled rate, % (95% CI)	*I* ^2^	Pooled rate, % (95% CI)		*I* ^2^
Positivity rates of high-risk individuals in EC screening		—^a^	—	44.12 (28.76-60.69)	100%	23.11 (19.52-27.13)		100%
Compliance rates of endoscopy examinations		50.11 (36.65-63.58)	100%	52.40 (44.58-60.22）	100%	23.67 (18.24-29.10)		100%
**Endoscopy findings**								
	Negative endoscopy findings	94.72 (92.82-96.14)	100%	96.93 (92.99-98.68)	99%	99.41 (99.07-99.62)		82%
	LGIN^b^	3.99 (2.78-5.69)	100%	2.55 (1.03-6.19)	99%	0.34 (0.14-0.81)		84%
	Positive cases	1.03 (0.82-1.30)	98%	0.48 (0.25-0.93)	98%	0.12 (0.07-0.21)		46%
	Early detection rates	82.09 (74.85-87.59)	93%	80.76 (69.24-88.68)	93%	80.08 (27.21-97.74)		85%

^a^Not applicable.

^b^LGIN: low-grade intraepithelial neoplasia.

#### Pooled Rates of Endoscopy Findings

The prevalence of endoscopy findings varied widely by region. Specifically, the pooled detection rates of positive cases decreased from high-risk areas to rural China and urban China (Table 3, Figure S2 in Multimedia Appendix 1). The pooled detection rate of LGIN was also in the same order, being the highest in high-risk areas, followed by rural China and urban China, as shown in Table 3 and Figure S3 in Multimedia Appendix 1. Consequently, the detection rates of negative endoscopy findings increased from high-risk areas to rural China and urban China (Table 3, Figure S4 in Multimedia Appendix 1).

#### Pooled Rates of Early Detection Among Positive Cases

A total of 34 of the 44 included studies reported early detection cases, covering 8445 patients diagnosed with positive cases at endoscopy screening (Figure S5 in Multimedia Appendix 1). The pooled early detection rate was approximately 81.90% (95% CI 75.58%-86.88%), which was similar to the rates in high-risk areas, rural China, and urban China (Table 3).

#### Pooled Detection Rates of Endoscopy Findings by Subgroups

Table 4 summarizes the pooled rates of endoscopy findings of sex-specific and age-specific subgroup analyses. Overall, males had higher detection rates of positive cases (1.65% vs 0.82%) and LGIN (5.71% vs 3.66%) and a lower detection rate of negative endoscopy findings (92.33% vs 95.38%) than females. The detection rates of positive cases and LGIN increased with age group, as the rates of positive cases were 0.52% in the 40-49 years group, 1.26% in the 50-59 years group, and 2.73% in the 60-69 years group, and the corresponding rates of LGIN were 2.62%, 4.24%, and 7.13%, respectively. The sex-specific and age-group detection rates of endoscopy screening in the three specific areas are also shown in Table 4, demonstrating the highest detection rates of positive cases and LGIN in high-risk areas, followed by rural China and urban China in each subgroup.

**Table 4:** Prevalence of negative endoscopy findings, low-grade intraepithelial neoplasia (LGIN), and positive cases in different subgroups in population-based esophageal cancer screening in China.

Characteristics				Studies, n		Study participants, n		Negative endoscopy findings					LGIN					Positive cases		
								Pooled prevalence rate, % (95% CI)		*I* ^2^		Pooled prevalence rate, % (95% CI)			*I* ^2^		Pooled prevalence rate, % (95% CI)			*I* ^2^
**All**																				
	**Sex**																			
		Male	17		165,522		92.33 (89.03-94.69)		100%		5.71 (3.71-8.69)			99%		1.65 (1.22-2.24)			97%	
		Female	17		214,180		95.38 (92.82-97.06)		100%		3.66 (2.22-5.98)			100%		0.82 (0.55-1.20)			97%	
	**Age group (years)**																			
		40-49	12		104,222		96.57 (93.33-98.26)		100%		2.62 (1.42-4.79)			99%		0.52 (0.22-1.23)			100%	
		50-59	12		105,033		94.04 (89.40-96.72)		100%		4.24 (2.17-8.11)			100%		1.26 (0.71-2.21)			98%	
		60-69	12		90,413		89.10 (82.14-93.56)		100%		7.13 (4.00-12.40)			99%		2.73 (1.57-4.71)			99%	
**High-risk areas**																				
	**Sex**																			
		Male	13		124,774		91.04 (86.81-94.00)		100%		6.60 (4.08-10.49)			100%		1.99 (1.46-2.71)			96%	
		Female	13		157,326		94.40 (91.13-96.51)		100%		4.39 (2.56-7.41)			100%		0.95 (0.64-1.42)			97%	
	**Age group (years)**																			
		40-49	8		73,745		94.79 (88.78-97.66)		100%		3.80 (1.92-7.40)			99%		0.80 (0.26-2.48)			100%	
		50-59	8		68,611		91.81 (84.34-95.89)		100%		5.46 (2.41-11.89)			100%		2.05 (1.19-3.50)			98%	
		60-69	8		59,762		84.81 (75.09-91.18)		100%		9.17 (4.62-17.40)			100%		4.30 (2.50-7.30)			99%	
**Rural China**																				
	**Sex**																			
		Male	3		58,943		93.63 (92.09-94.88)		98%		5.42 (4.58-6.42)			96%		0.92 (0.56-1.50)			97%	
		Female	3		81,694		96.05 (95.10-96.82)		98%		3.47 (2.96-4.06)			95%		0.45 (0.24-0.83)			98%	
	**Age group (years)**																			
		40-49	3		46,472		97.93 (97.70-98.14)		63%		1.86 (1.69-2.04)			47%		0.21 (0.15-0.32)			74%	
		50-59	3		52,811		95.22 (94.54-95.81)		92%		4.26 (3.93-4.61)			74%		0.50 (0.29-0.85)			95%	
		60-69	3		41,339		92.02 (89.52-93.96)		99%		6.69 (5.34-8.36)			98%		1.24 (0.71-2.15)			98%	
**Urban China**																				
	**Sex**																			
		Male	1		479		98.75 (97.24-99.44)		—^a^		0.63 (0.20-1.92)			—		0.63 (0.20-1.92)			—	
		Female	1		672		99.70 (98.82-99.93)		—		0.15 (0.02-1.05)			—		0.15 (0.02-1.05)			—	
	**Age group (years)**																			
		40-49	1		347		99.71 (97.98-99.96)		—		0			—		0.29 (0.04-2.02)			—	
		50-59	1		394		99.49 (97.99-99.87)		—		0.25 (0.04-1.78)			—		0.25 (0.04-1.78)			—	
		60-69	1		409		98.78 (97.10-99.49)		—		0.73 (0.24-2.25)			—		0.49 (0.12-1.93)			—	

^a^Not applicable.

### Validation of the Meta-analysis Results

#### Sensitivity Analysis

To assess whether publication year, sample size, and the number of study centers had a dominant effect on the meta-analysis, we additionally analyzed their effect on the detection of positive cases (see Table S8 of Multimedia Appendix 1). The findings in this analysis showed that studies with publication years before 2009 and sample sizes less than 5000 had a significantly higher detection rate of positive cases in high-risk areas. In addition, multicenter studies presented lower heterogeneity in urban China.

#### Heterogeneity

High heterogeneity (*I*^2^) among studies was observed for all estimated indices, and these findings remained unchanged in the subgroups (Table 4; Table S8 in Multimedia Appendix 1).

#### Publication Bias

Publication bias assessment was performed based on the prevalence of positive cases. Visual inspection of the funnel plots (Figure S6 in Multimedia Appendix 1) and the two-tailed Egger test (*P*=.10) demonstrated no evidence of publication bias.

## Discussion

Based on this systematic review and meta-analysis of 66 studies covering over 2 million asymptomatic Chinese populations participating in population-based EC screening, we summarized a series of crucial indices to profile the status of EC screening in China. Under the current EC screening practice, the positivity rates of the high-risk population and compliance rates with endoscopic examinations, yields from EC screening, and burden of surveillance endoscopy varied greatly in populations from high-risk areas and other nonhigh-risk areas (rural China and urban China). These findings not only show the actual situation of current population-based ESCC screening in China but also reflect corresponding challenges and potential future directions in scientific research and policy-making to promote EC prevention in China.

Identifying high-risk individuals for EC to undergo further endoscopic examinations has been one of the most critical factors for the success of ESCC screening programs. Selection of assessment items and the threshold to define endoscopic examination eligibility are essential but troublesome tasks in this field [75]. In nonhigh-risk areas of EC where the RSES strategy was adopted, the pooled positivity rate of high-risk individuals for endoscopic examinations in the rural Chinese population was approximately twice that in the urban Chinese population (44.12% vs 23.11%), fully reflecting the rural-urban inequality of exposure to EC risk factors. However, the existing individualized assessment tool in EC screening programs or trials in China differed in the assessment items, thresholds, and definition of outcomes (EC or UGI cancer), as summarized in Table S5 of Multimedia Appendix 1. Few assessment tools have estimated the accuracy and effectiveness of EC screening. In addition, all the existing prediction models for EC presented a high risk of bias and none of these models was estimated in diverse populations, which limited the implementation values in large-scale population-based EC screening programs or even the national EC screening program [75]. Before implementing the national EC screening program, development of a specific high-risk assessment strategy to define eligible individuals for endoscopy screening should be prioritized. Through comprehensive estimation and validation in diverse populations with national representatives, updated individualized EC risk assessment strategies could be developed in the future, serving EC screening in China and other countries.

Optimal compliance for endoscopic examinations in eligible asymptomatic individuals is essential to confer benefits from EC screening, as shown in cohort and modeling studies [6,76,77]. However, the overall compliance of endoscopy screening in existing population-based EC screening programs or trials in China was found to be suboptimal, especially in the urban Chinese population, with a compliance rate of less than 24%. Therefore, improving the endoscopic compliance rate by adopting comprehensive strategies is an urgent need for EC screening programs. Several available measures could be taken in the future. First, from the aspect of providers of EC screening programs, there is an urgent need to conduct multifaceted actions to promote and improve the core knowledge of cancer prevention and control in Chinese residents and help them correctly understand cancer and prevent it [78]. A previous EC screening study among 28,543 high-risk individuals for EC showed a positive association between cancer prevention awareness and compliance with endoscopic examinations [79]. This highlights the importance and feasibility of raising eligible individuals’ awareness of cancer prevention in improving compliance rates in population-based EC screening programs. Second, for community servers in population-based EC screening, more attention and interventions should be paid to several vulnerable populations in health literacy to improve their compliance rate with endoscopic examinations. These populations included males, cigarette smokers, and those with low socioeconomic status, who had a higher risk of developing EC but lower compliance rates of endoscopic examinations, as observed in previous population-based EC screening studies [31,33,79]. In addition, optimization of the screening procedure, such as shortening the waiting time for endoscopic examinations, may be considered another effective measure to improve compliance, as indicated in population-based cancer screening programs [80].

The endoscopy findings exhibited apparent disparities among studies, mainly due to the heterogeneous study populations in the disease burden of EC, different EC screening strategies, and varying compliance rate for endoscopic examination, which were mentioned above. Specifically, a higher screening benefit was obtained in high-risk areas, followed by rural China and urban China, with pooled prevalence rates of positive cases being 1.03%, 0.48%, and 0.12%, respectively. In addition, early detection among positive cases was more than 80% in all studies, which emphasizes the importance of EC screening in improving survival and quality of life. In terms of the surveillance endoscopy burden, a higher burden of LGIN was also observed in high-risk areas, followed by rural China and urban China, with pooled rates of 3.99%, 2.55%, and 0.34%, respectively. A much larger fraction of the baseline endoscopy screening population comprised those diagnosed with negative endoscopy findings, with prevalence higher than 90% in all previous reports. These results will help policy makers grasp the profile of population-based EC screening under the framework of current strategies in different regions and provide essential benchmarks for health economic evaluation models or other studies. Moreover, there is a considerable need for managing the vast population identified with negative endoscopy findings and LGIN [7]. The current Chinese guidelines for EC screening recommend surveillance intervals of 1-3 years for patients with LGIN and 5 years for individuals with negative endoscopy findings [81]. However, these recommendations were developed based on limited studies with different findings on observational or modeling studies from ESCC high-risk areas in China [76,77,82-84]. More high-quality studies are urgently needed to estimate and update these recommendations.

To the best of our knowledge, this is the first study to provide a comprehensive profile of the available research on population-based EC screening in the Chinese population, which offers comprehensive and objective evidence for policy makers. Findings in this study have important implications for better conducting EC screening in the future. First, development of high-risk assessment criteria for selecting eligible individuals for endoscopic examinations for diverse populations should be prioritized. Second, multidimensional attempts are urgently needed to improve compliance with endoscopic examinations, including improving residents’ cancer literacy and optimizing and simplifying screening procedures. Third, more robust and high-quality cohort and modeling studies are urgently needed to estimate or optimize the current surveillance strategy, which will essentially compensate for the limited evidence in the Chinese population.

Our study also has several limitations. First, significant heterogeneity was observed in all pooled indices. Understandably, high heterogeneity is unavoidable given that study-related factors (screening strategy and population-based disease burden of EC) and individual-related factors (individualized risk of EC, compliance with endoscopic examinations, age, and sex) could not be controlled for in all studies. In this meta-analysis, we performed several subgroup analyses with consideration of these significant factors contributing to heterogeneity. Second, a detailed comparison of exposure factors related to developing EC and determinations of compliance with endoscopic examinations in rural-urban Chinese populations were not performed due to data limitations in the existing studies. Third, subgroup analysis of endoscopy findings was limited to sex-specific and age-specific findings. Other potential essential factors, including individual-related (such as smoking status, family history of cancer, and disease history of the digestive system) and procedure-related (such as endoscopist and pathologist performance and quality of endoscopic examinations) factors, were limited due to the lack of data. In addition, available studies and data under different EC screening strategies need to be more balanced. Data on endoscopy findings in high-risk areas were sufficient; however, data to profile EC screening in nonhigh-risk areas were limited. There were only four studies in rural China that reported compliance with endoscopy, and only 18,421 individuals with endoscopy screening were reported in the urban Chinese population. These limited data could not comprehensively profile actual population-based EC screening in this population, and an updated meta-analysis will be performed when more evidence is available.

In summary, in this meta-analysis, the current profile of population-based EC screening in China and potential challenges were summarized with a series of indices in screening procedures (positivity rates of high-risk individuals, compliance rates, and endoscopic findings) by different regions. Under the current screening framework, a higher screening benefit was observed in high-risk areas than in other regions. To promote EC screening and reduce the current inequality of screening in China, more focus should be given to optimizing strategies of high-risk individual assessment and surveillance management to improve compliance with endoscopic examination.

## Appendix

Multimedia Appendix 1 Supporting methods and results.

## Abbreviations

EC: esophageal cancer
ESCC: esophageal squamous cell carcinoma
LGIN: low-grade intraepithelial neoplasia
MeSH: Medical Subject Headings
PRISMA: Preferred Reporting Items for Systematic Review and Meta-Analyses
PROSPERO: International Prospective Register of Systematic Reviews
RSES: risk-stratified endoscopic screening
UGI: upper gastrointestinal

## Data Availability

The data sets generated and/or analyzed during this study are available from the corresponding author on reasonable request.
