# Supplementary material for: Profiles and Findings of Population-Based Esophageal Cancer Screening With Endoscopy in China: Systematic Review and Meta-analysis
Source: JMIR Public Health Surveill. 2023 Jun 1;9:e45360. doi: 10.2196/45360 (PMC10273033; doi:10.2196/45360)
Supplement: Multimedia Appendix 1 [file publichealth_v9i1e45360_app1.docx]

Table S1: Full search strategy of the meta-analysis

| **Database** | **Search strategy** | |
| --- | --- | --- |
| **PubMed** | #1 | ((esophageal neoplasms[MeSH Terms]) OR (esophag*[tiab]) OR (upper gastrointestinal[tiab])) |
|  | #2 | ((cancer [tiab]) OR (neoplasm*[tiab]) OR (tumor*[tiab]) OR (tumour*[tiab]) OR (carcinoma*[tiab])) |
|  | #3 | screening[tiab] |
|  | #4 | ((China[MeSH Terms]) OR (China[tiab]) OR (Chinese[tiab]) OR (Taiwan*[tiab]) OR (Hongkong[tiab]) OR (Macau[tiab])) |
|  | #5 | ("1000/01/01"[Date - Publication] : "2022/12/31"[Date - Publication]) |
|  | #6 | #1 AND #2 AND #3 AND #4 AND #5 |
| **Embase** | #1 | esophagus tumor/exp OR esophag*:ab, ti OR upper gastrointestinal:ab, ti |
|  | #2 | cancer:ab, ti OR neoplasm*:ab, ti OR tumor*:ab, ti OR tumour*:ab, ti OR carcinoma*:ab, ti |
|  | #3 | screening:ab, ti |
|  | #4 | (China/exp OR China:ab, ti OR chinese:ab, ti OR taiwan*:ab, ti OR Hongkong*:ab, ti OR Macau*:ab, ti) AND [<1966-2022]/py |
|  | #5 | #1 AND #2 AND #3 AND #4 |
| **Wanfang** | 题名或关键词: (("食管" or "上消化道") and ("癌" or "肿瘤") and ("筛查" or "早诊早治")) or 摘要:(("食管" or "上消化道") and ("癌" or "肿瘤") and ("筛查" or "早诊早治"))  **Translation:**  Title or Key words: (("esophagus" or "upper gastrointestinal tract") and ("cancer" or "tumor") and ("screening" or "early diagnosis and early treatment")) or Abstract:(("esophagus" or "upper gastrointestinal tract") and ("cancer" or "tumor") and ("screening" or "early diagnosis and early treatment")) | |
| **CNKI** | (TI=食管 or AB=食管 or TI=上消化道 or AB=上消化道) and (TI=癌 or TI=肿瘤 or AB=癌 or AB=肿瘤) and (TI=筛查 or TI=早诊早治 or AB=筛查 or AB=早诊早治)  **Translation:**  (TI=esophagus or AB=esophagus or TI=upper gastrointestinal tract or AB=upper gastrointestinal tract) and (TI=cancer or TI=tumor or AB=cancer or AB=tumor) and (TI=screening or TI= early diagnosis and early treatment or AB=screening or AB= early diagnosis and early treatment) | |

Abbreviation: CNKI, China National Knowledge Infrastructure.

Table S2: Quality appraisal tool for population-based EC screening studies

*This questionnaire was adapted from Hoy et al. J Clin Epidemiol. 2012* [1]*.*

| **Risk of bias item** | **Criteria for answers (circle**  **one option)** | **Additional notes and examples** |
| --- | --- | --- |
| ***External validity*** |  |  |
| 1. Was the study’s target population a Close representation of the national population in relation to relevant variables, e.g. age, sex, occupation? | - Yes (LOW RISK): The study’s target population was a close representation of the national population. - No (HIGH RISK): The study’s target population was clearly NOT representative of the national population. | The target population refers to the group of people or entities to which the results of the study will be generalized. Examples:   - The study was a national health survey of people 15 years and over and the sample was drawn from a list that included all individuals in the population aged 15 years and over. The answer is: Yes (LOW RISK). - The study was conducted in one province only, and it is not clear if this was representative of the national population. The answer is: No (HIGH RISK). - The study was undertaken in one village only and it is clear this was not representative of the national population. The answer is: No (HIGH RISK). |
| 2. Was the sampling frame a true or close representation of the target population? | - Yes (LOW RISK): The sampling frame was a true or close representation of the target population. - No (HIGH RISK): The sampling frame was NOT a true or close representation of the target population. | The sampling frame is a list of the sampling units in the target population and the study sample is drawn from this list. Examples:   - The sampling frame was a list of almost every individual within the target population. The answer is: Yes (LOW RISK). - The cluster sampling method was used and the sample of clusters/villages was drawn from a list of all villages in the target population. The answer is: Yes (LOW RISK). - The sampling frame was a list of just one particular ethnic group within the overall target population, which comprised many groups. The answer is: No (HIGH RISK). |
| 3. Was some form of random selection used to select the sample, OR, was a census undertaken? | - Yes (LOW RISK): A census was undertaken, OR, some form of random selection was used to select the sample (e.g. simple random sampling, stratified random sampling, cluster sampling, systematic sampling). - No (HIGH RISK): A census was NOT undertaken, AND some form of random selection was NOT used to select the sample. | A census collects information from every unit in the sampling frame. In a survey, only part of the sampling frame is sampled. In these instances, random selection of the sample helps minimize study bias. Examples:   - The sample was selected using simple random sampling. The answer is: Yes (LOW RISK). - The target population was the village and every person in the village was sampled. The answer is: Yes (LOW RISK). - The nearest villages to the capital city were selected in order to save on the cost of fuel. The answer is: No (HIGH RISK). |
| 4. Was the likelihood of  non-response bias minimal? | - Yes (LOW RISK): The response rate for the study was >/=75%, OR, an analysis was performed that showed no significant difference in relevant demographic characteristics between responders and non responders - No (HIGH RISK): The response rate was <75%, and if any analysis comparing responders and non-responders was done, it showed a significant difference in relevant demographic characteristics between responders and non-responders. | Examples:   - The response rate was 68%; however, the researchers did an analysis and found no significant difference between responders and non-responders in terms of age, sex, occupation and socioeconomic status. The answer is: Yes (LOW RISK). - The response rate was 65% and the researchers did NOT carry out an analysis to compare relevant demographic characteristics between responders and non-responders. The answer is: No (HIGH RISK). - The response rate was 69% and the researchers did an analysis and found a significant difference in age, sex and socioeconomic status between responders and non-responders. The answer is: No (HIGH RISK). |
| ***Internal validity*** |  |  |
| 5. Were data collected  directly from the subjects (as opposed to  a proxy)? | - Yes (LOW RISK): All data were collected directly from the subjects. - No (HIGH RISK): In some instances, data were collected from a proxy. | A proxy is a representative of the subject. Examples:   - All eligible subjects in the household were interviewed separately. - The answer is: Yes (LOW RISK). - A representative of the household was interviewed and questioned about the presence of low back pain in each household member. - The answer is: No (HIGH RISK). |
| 6. Was an acceptable case  definition used in the study? | - Yes (LOW RISK): An acceptable case definition was used. - No (HIGH RISK): An acceptable case definition was NOT used. | - For a study on low back pain, the following case definition was used: “Low back pain is defined as activity-limiting pain lasting more than one day in the area on the posterior aspect of the body from the bottom of the 12th rib to the lower gluteal folds.” The answer is: Yes (LOW RISK). - For a study on back pain, there was no description of the specific anatomical location “back” referred to. The answer is: No (HIGH RISK). - For a study on osteoarthritis, the following case definition was used: “Symptomatic osteoarthritis of the hip or knee, radiologically confirmed as Kellgren-Lawrence grade 2-4”. The answer is: LOW RISK. |
| 7. Was the study instrument that measured the parameter of  interest (e.g. prevalence of low back pain)  shown to have reliability and validity (if necessary)? | - Yes (LOW RISK): The study instrument had been shown to have reliability and validity (if this was necessary), e.g. test-retest, piloting, validation in a previous study, etc. - No (HIGH RISK): The study instrument had NOT been shown to have reliability or validity (if this was necessary). | - The authors used the COPCORD questionnaire, which had previously been validated. They also tested the interrater reliability of the questionnaire. The answer is: Yes (LOW RISK). - The authors developed their own questionnaire and did not test this for validity or reliability. The answer is: No (HIGH RISK). |
| 8. Was the same mode of data collection used for all subjects? | - Yes (LOW RISK): The same mode of data collection was used for all subjects. - No (HIGH RISK): The same mode of data collection was NOT used for all subjects. | The mode of data collection is the method used for collecting information from the subjects. The most common modes are face-to face interviews, telephone interviews and self-administered questionnaires. Examples:   - All eligible subjects had a face-to-face interview. The answer is: Yes (LOW RISK). - Some subjects were interviewed over the telephone and some filled in postal questionnaires. The answer is: No (HIGH RISK). |
| 9. Was the length of the shortest prevalence  period for the parameter of interest appropriate? | - Yes (LOW RISK): The shortest prevalence period for the parameter of interest was appropriate (e.g. point prevalence, one-week prevalence, one-year prevalence). - No (HIGH RISK): The shortest prevalence period for the parameter of interest was not appropriate (e.g. lifetime prevalence) | The prevalence period is the period that the subject is asked about e.g. “Have you experienced low back pain over the previous year?” In this example, the prevalence period is one year. The longer the prevalence period, the greater the likelihood of the subject forgetting if they experienced the symptom of interest (e.g. low back pain). Examples:   - Subjects were asked about pain over the past week. The answer is: Yes (LOW RISK). - Subjects were only asked about pain over the past three years. The answer is: No (HIGH RISK). |
| 10. Were the numerator(s) and denominator(s) for the parameter of interest appropriate? | - Yes (LOW RISK): The paper presented appropriate numerator(s) AND denominator(s) for the parameter of interest (e.g. the prevalence of low back pain). - No (HIGH RISK): The paper did present numerator(s) AND denominator(s) for the parameter of interest but one or more of these were inappropriate. | There may be errors in the calculation and/or reporting of the  numerator and/or denominator. Examples:   - There were no errors in the reporting of the numerator(s) AND denominator(s) for the prevalence of low back pain. The answer is: Yes (LOW RISK). - In reporting the overall prevalence of low back pain (in both men and women), the authors accidentally used the population of women as the denominator rather than the combined population. The answer is: No (HIGH RISK). |
| ***11. Summary item on the overall risk of study bias*** | | |
| - LOW RISK OF BIAS: Further research is very unlikely to change our confidence in the estimate. - MODERATE RISK OF BIAS: Further research is likely to have an important impact on our confidence in the estimate and may change the estimate. - HIGH RISK OF BIAS: Further research is very likely to have an important impact on our confidence in the estimate and is likely to change the estimate. | | |

Abbreviation: EC, esophageal cancer.

Table S3: The definitions of outcomes in this meta-analysis

| Outcomes | Definition |
| --- | --- |
| Positivity rate of high-risk individuals | The numbers of individuals assessed as high-risk population for EC/UGI cancer divided by the numbers of participating individualized risk assessment. |
| Compliance rates to endoscopy examination | The number of undergoing endoscopy examinations divided by the number of high-risk populations. |
| Detection rates of endoscopy findings | The numbers of specific lesions divided by the numbers of endoscopies. |
| Early detection rate | The numbers of severe dysplasia, carcinoma in situ, and submucosal carcinoma divided by the numbers of positive cases. |

Abbreviations: EC, esophageal cancer; UGI, upper gastrointestinal.

Table S4: Characteristics of 66 population-based EC screening studies included in this meta-analysis

| Study | Region | | Screening strategy | No. of study centers | Study period | Eligible age, years | Proportion of men | No. of invited to EC screening | No. of high-risk individuals | No. of undergoing endoscopy examination | No. of Endoscopy findings | | | No. of cases with early detection |
| --- | --- | --- | --- | --- | --- | --- | --- | --- | --- | --- | --- | --- | --- | --- |
|  | Province | Type |  |  |  |  |  |  |  |  | Positive cases | LGIN | Negative endoscopy findings |  |
| Lu XJ; 2003 [2] | Hebei | High-risk areas | Universal endoscopy screening | 1 | 2001-2002 | 40-69 | 48.34 | 2725 | / | 2013 | 121 | 329 | 1563 | 107 |
| Wang XZ; 2009 [3] | Shanxi | High-risk areas | Universal endoscopy screening | 1 | 2006-2008 | 40-69 | 49.81 | NA | / | 5073 | 45 | 406 | 4622 | 33 |
| Yin C; 2009 [4] | Shandong | High-risk areas | Universal endoscopy screening | 1 | 2004-2006 | 40-69 | 44.26 | NA | / | 9536 | 161 | 490 | 8885 | 161 |
| Wen DG; 2009 [5] | Hebei | High-risk areas | Universal endoscopy screening | 1 | 2001-2005 | 40-69 | NA | 2103 | / | 1514 | NA | NA | NA | NA |
| Chen LL; 2011 [6] | Gansu | High-risk areas | Universal endoscopy screening | 2 | 2009 | 40-69 | NA | NA | / | 2050 | 12 | 10 | 2028 | 1 |
| Zou WD; 2011 [7] | Hebei | High-risk areas | Universal endoscopy screening | 1 | 2001-2008 | 40-69 | 51.77 | NA | / | 4124 | 96 | 397 | 3631 | 82 |
| Zhang YZ; 2012 [8] | Shanxi | High-risk areas | Universal endoscopy screening | 1 | 2006-2010 | 40-69 | 49.73 | 10,028 | / | 7094 | 134 | 1021 | 5939 | 93 |
| Guo S; 2013 [9] | Hebei | High-risk areas | Universal endoscopy screening | 3 | 2005-2010 | 40-69 | 48.69 | NA | / | 23,561 | 261 | 2145 | 21,155 | 191 |
| Wang ZQ; 2014 [10] | Sichuan | High-risk areas | Universal endoscopy screening | 8 | 2010-2013 | 40-69 | 51.03 | 1,359,600 | / | 6571 | 66 | 501 | 6004 | NA |
| Yang L; 2014 [11] | Hebei | High-risk areas | Universal endoscopy screening | 1 | 2011-2014 | 40-69 | NA | NA | / | 2102 | 11 | 26 | 2065 | 9 |
| Zhang ZY; 2014 [12] | Gansu | High-risk areas | Universal endoscopy screening | 1 | 2009-2012 | 40-69 | 47.26 | NA | / | 8007 | 43 | 219 | 7745 | 38 |
| Du LB; 2015 [13] | Zhejiang | High-risk areas | Universal endoscopy screening | 2 | 2010-2013 | 40-69 | NA | NA | / | 9037 | 44 | 468 | 8525 | 40 |
| Liang SY; 2015 [14] | Shandong | High-risk areas | Universal endoscopy screening | 1 | 2006-2012 | 40-69 | 42.57 | NA | / | 24,759 | 340 | 1781 | 22,638 | 288 |
| Lv CH; 2015 [15] | Jiangxi | High-risk areas | Universal endoscopy screening | 1 | 2010-2013 | 40-69 | 51.83 | NA | / | 6000 | 18 | 85 | 5897 | 16 |
| Tuo JY; 2015 [16] | Hubei | High-risk areas | Universal endoscopy screening | 5 | 2011-2013 | 40-69 | NA | NA | / | 8052 | 73 | 195 | 7784 | 40 |
| Zhang M; 2015 [17] | Henan | High-risk areas | Universal endoscopy screening | 12 | 2009-2013 | 40-69 | NA | NA | / | 88,263 | 1210 | 7224 | 79,829 | 1028 |
| Wei WQ; 2015 [18] | Hebei | High-risk areas | Universal endoscopy screening | 1 | 1999-2000 | 40-69 | 49.08 | 6827 | / | 3319 | 201 | 578 | 2540 | NA |
| Zheng XZ; 2015 [19] | Jiangsu | High-risk areas | Universal endoscopy screening | 1 | 2006-2012 | 40-69 | 42.83 | NA | / | 12,453 | 106 | 143 | 12,204 | 104 |
| Li ZH; 2016 [20] | Hubei | High-risk areas | Universal endoscopy screening | 1 | 2012-2014 | 40-69 | 64.78 | NA | / | 6000 | 37 | 68 | 5895 | NA |
| Qian F; 2016 [21] | Henan | High-risk areas | Universal endoscopy screening | 1 | 2010-2015 | 40-69 | 38.31 | NA | / | 23,513 | 124 | 249 | 23,140 | 70 |
| Wang W; 2016 [22] | Zhejiang | Urban China | Endoscopy for high-risk individuals | 2 | 2014-2015 | 40-69 | NA | 47,827 | 47,827 | 2127 | NA | NA | NA | NA |
| Wang M; 2016 [23] | Hebei, Shandong, Henan | High-risk areas | Universal endoscopy screening | 3 | 2005-2009 | 40-69 | 46.57 | 46,568 | / | 21,764 | 473 | 3163 | 18,128 | NA |
| He X;  2017 [24] | Sichuan | High-risk areas | Universal endoscopy screening | 1 | 2016-2017 | 40-69 | 41.55 | NA | / | 2000 | 51 | 187 | 1762 | NA |
| He YL; 2017 [25] | Gansu | High-risk areas | Universal endoscopy screening | 1 | 2011-2015 | 40-69 | NA | NA | / | 5838 | 57 | 99 | 5682 | 45 |
| Jing YH; 2017 [26] | Sichuan | High-risk areas | Universal endoscopy screening | 1 | 2011-2015 | 40-69 | NA | 32,719 | / | 14,000 | NA | NA | NA | NA |
| Wang X; 2017 [27] | Sichuan | High-risk areas | Universal endoscopy screening | 10 | 2006-2014 | 40-69 | 42.61 | NA | / | 105,561 | 1193 | 5841 | 98,527 | 900 |
| Xu CL; 2017 [28] | Jiangsu | Urban China | Endoscopy for high-risk individuals | 1 | 2012-2015 | >35 | 46.35 | 13,037 | 2260 | 1065 | NA | NA | NA | NA |
| Xu H;  2017 [29] | Jiangsu | Urban China | Endoscopy for high-risk individuals | 1 | 2016 | 40-69 | 46.14 | 25,056 | 6909 | 117 | NA | NA | NA | NA |
| Zhang N; 2017 [30] | Shandong | High-risk areas | Universal endoscopy screening | 9 | 2013-2016 | 40-69 | NA | NA | / | 107,431 | 1093 | 4471 | 101,867 | 941 |
| Zhou TH; 2017 [31] | Xinjiang | Urban China | Endoscopy for high-risk individuals | 1 | 2015 | 40-69 | 41.54 | NA | NA | 1548 | 3 | 1 | 1544 | NA |
| Zhu JY; 2017 [32] | Xinjiang | Urban China | Endoscopy for high-risk individuals | 1 | 2016 | 40-69 | NA | 50,711 | 13,911 | 1920 | NA | NA | NA | NA |
| Ding GH; 2018 [33] | Gansu | High-risk areas | Universal endoscopy screening | 4 | 2012-2016 | 40-69 | 48.89 | NA | / | 6392 | 59 | 441 | 5892 | 54 |
| Liu YZ; 2018 [34] | Jiangsu | Urban China | Endoscopy for high-risk individuals | 1 | 2014-2016 | 40-69 | 43.87 | 70,270 | 10,543 | 2537 | NA | NA | NA | NA |
| Sun GF; 2018 [35] | Xinjiang | Urban China | Endoscopy for high-risk individuals | 1 | 2014 | 40-69 | 46.37 | 49,574 | 10,105 | 2003 | NA | NA | NA | NA |
| Tang X; 2018 [36] | Sichuan | High-risk areas | Universal endoscopy screening | 18 | 2012-2016 | 40-69 | 41.55 | NA | / | 10,102 | 179 | 535 | 9388 | 155 |
| Cao XQ; 2019 [37] | Henan | Rural China | Endoscopy for high-risk individuals | 3 | 2007-2015 | 40-69 | 39.58 | NA | NA | 23,733 | 94 | 902 | 22,737 | 67 |
| Huang YL; 2019 [38] | Guangxi | Urban China | Endoscopy for high-risk individuals | 2 | 2016-2017 | 40-69 | 38.64 | NA | NA | 2399 | 3 | 17 | 2379 | 1 |
| Liang F; 2019 [39] | Jiangsu | High-risk areas | Universal endoscopy screening | 1 | 2014-2016 | 40-69 | 50.48 | NA | / | 1983 | 29 | 318 | 1636 | 24 |
| Lin YP; 2019 [40] | Yunnan | Urban China | Endoscopy for high-risk individuals | 1 | 2015-2018 | 40-74 | 32.90 | 95,296 | 25,300 | 6158 | 3 | 31 | 6124 | NA |
| Ma HM; 2019 [41] | Shandong | Rural China | Endoscopy for high-risk individuals | 5 | 2007-2015 | 40-69 | 42.26 | NA | NA | 41,068 | 217 | 1605 | 39,246 | 165 |
| Wang WP; 2019 [42] | Hebei | High-risk areas | Universal endoscopy screening | 1 | 2005-2018 | 40-69 | 44.75 | NA | / | 27,994 | 333 | 2486 | 25,175 | 293 |
| Xiao HF; 2019 [43] | Hunan | Urban China | Endoscopy for high-risk individuals | 1 | 2012-2018 | 40-74 | 49.87 | 293,760 | 39,111 | 9926 | NA | NA | NA | NA |
| Zhang ZY; 2019 [44] | Gansu | High-risk areas | Universal endoscopy screening | 1 | 2009-2017 | 40-69 | NA | 38,400 | / | 12,900 | NA | NA | NA | NA |
| Guo LW; 2019 [45] | Henan | Urban China | Endoscopy for high-risk individuals | 3 | 2013-2017 | 40-74 | NA | 179,002 | 43,423 | 7996 | NA | NA | NA | NA |
| He ZH; 2019 [46] | Henan | High-risk areas | Universal endoscopy screening | 1 | 2012-2016 | 45-69 | NA | NA | / | 15,188 | 113 | 560 | 14,515 | 79 |
| Liu MF; 2019 [47] | Henan | High-risk areas | Universal endoscopy screening | 1 | 2007-2013 | 25-65 | 49.15 | 6405 | / | 5632 | NA | NA | NA | NA |
| Feng X; 2020 [48] | Jiangsu | High-risk areas | Universal endoscopy screening | 1 | 2011-2017 | 40-69 | 43.64 | NA | / | 14,687 | 77 | 341 | 14,269 | 60 |
| Jia SC; 2020 [49] | Anhui | High-risk areas | Universal endoscopy screening | 10 | 2018-2019 | 40-69 | 52.62 | NA | / | 29,386 | 296 | 191 | 28,899 | 157 |
| Liu YY; 2020 [50] | Shenyang | Urban China | Endoscopy for high-risk individuals | 1 | 2016-2019 | 40-74 | NA | 102,913 | 25,319 | 4406 | NA | NA | NA | NA |
| Shi J;  2020 [51] | Hebei | Urban China | Endoscopy for high-risk individuals | 2 | 2018-2019 | 40-74 | 44.40 | 37,849 | 9222 | 1838 | 4 | 1 | 1833 | NA |
| Sun Q; 2020 [52] | Jiangsu | Rural China | Endoscopy for high-risk individuals | 3 | 2009-2017 | 40-69 | 42.72 | NA | NA | 31,650 | 392 | 1666 | 29,592 | 352 |
| Zhao RC; 2020 [53] | Guangdong | Urban China | Endoscopy for high-risk individuals | 1 | 2017-2019 | 40-74 | 41.91 | 6072 | 1564 | 489 | NA | NA | NA | NA |
| Zhao T; 2020 [54] | Shandong | High-risk areas | Universal endoscopy screening | 1 | 2006-2015 | 40-69 | 49.20 | 42,188 | / | 23,470 | NA | NA | NA | NA |
| Zhu ZH; 2020 [55] | Guangxi | Urban China | Endoscopy for high-risk individuals | 1 | 2014-2017 | 40-74 | 44.75 | 152,630 | 66,400 | 5097 | NA | NA | NA | NA |
| Zhuang Y; 2020 [56] | Guizhou | High-risk areas | Universal endoscopy screening | 1 | 2018 | 40-69 | 33.69 | NA | / | 2048 | 11 | 6 | 2031 | 11 |
| Zeng HM; 2020 [57] | Henan, Jiangsu | Rural China | Endoscopy for high-risk individuals | 2 | 2015-2017 | 40-69 | 41.53 | 22,870 | 12,845 | 5962 | 11 | 30 | 5921 | NA |
|  | Hunan, Heilongjiang | Urban China | Endoscopy for high-risk individuals | 2 | 2015-2017 | 40-69 | 40.89 | 25,440 | 10,687 | 5327 | 4 | 61 | 5262 | NA |
|  | Hebei, Gansu, Henan | High-risk areas | Universal endoscopy screening | 3 | 2015-2017 | 40-69 | 43.07 | 62,123 | / | 26,633 | 239 | 1579 | 24,815 | 222 |
| Cao XQ; 2021 [58] | Henan | High-risk areas | Universal endoscopy screening | 16 | 2014-2019 | 40-69 | 41.25 | NA | / | 116,630 | 1232 | 7035 | 108,363 | 1057 |
| Lei RJ; 2021 [59] | Zhejiang | Urban China | Endoscopy for high-risk individuals | 1 | 2014-2018 | 40-74 | 44.27 | 50,114 | 13,151 | 3716 | NA | NA | NA | NA |
| Li J;  2021 [60] | Sichuan | High-risk areas | Universal endoscopy screening | 1 | 2006-2015 | 40-69 | NA | 120,655 | / | 42,340 | NA | NA | NA | NA |
| Tuo JY; 2021 [61] | Hubei | Urban China | Endoscopy for high-risk individuals | 3 | 2018-2019 | 40-74 | NA | 39,575 | 3800 | 1435 | NA | NA | NA | NA |
| Xiong WY; 2021 [62] | Jiangxi | Urban China | Endoscopy for high-risk individuals | 1 | 2018-2019 | 40-74 | 41.51 | 25,871 | 5365 | 1590 | NA | NA | NA | NA |
| Zhang J; 2021 [63] | Anhui | Urban China | Endoscopy for high-risk individuals | 1 | 2018 | 40-74 | 41.62 | NA | NA | 1151 | 4 | 4 | 1143 | NA |
| Zhang YZ; 2021 [64] | Shanxi | Urban China | Endoscopy for high-risk individuals | 3 | 2014-2018 | 40-74 | 46.77 | 184,539 | 41,441 | 6022 | NA | NA | NA | NA |
| Chen R; 2021 [65] | Hebei, Shandong, Henan, Shanxi, Sichuan, Jiangsu | High-risk areas | Universal endoscopy screening | 6 | 2005-2006 | 40-69 | 45.07 | 338,017 | / | 113,340 | 1438 | 9085 | 102,817 | NA |
| Li J;  2021 [66] | Jiangsu; Anhui; Shandong; Henan | Rural China | Endoscopy for high-risk individuals | 11 | 2010-2016 | 40-69 | 45.68 | 350,646 | 114,786 | 64,710 | NA | NA | NA | NA |
| Zhu L; 2022 [67] | Guizhou | High-risk areas | Universal endoscopy screening | 8 | 2009-2019 | 40-69 | NA | NA | / | 17666 | 29 | 65 | 14 | 13 |

Abbreviations: EC, esophageal cancer; LGIN, low-grade intraepithelial neoplasia; NA, not available.

Table S5: A summary of the current high-risk assessment tool or strategy in population-based endoscopy screening in China

| Study | Items in the high-risk assessment strategy | Definition of high-risk population | Assessment outcome in the strategy | Efficacy of the high-risk assessment tool | Type of applied population |
| --- | --- | --- | --- | --- | --- |
| Guo LW, 2019 [45] | smoking, alcohol drinking, tea drinking, dietary intake of pickled food, hot drink or hot food diet, indoor soot exposure in the past 10 years, history of upper gastrointestinal system diseases, body mass index, alcohol drinking, high‐salt diet, more‐dry diet, history of trauma, history of chronic gastritis, history of duodenal ulcer, and family history of esophageal or gastric cancer in first‐degree relative | NA.  Calculation by Harvard Risk Index, however, the specific definition of high-risk population was not reported. | Esophageal or gastric cancer | NA | Urban China |
| Zeng HM, 2020 [57] | Smoking at least 20 cigarettes per day and last for 10 years or more (1 score), Drinking at least 28 g ethanol per day and last for 10 years or more (1 score), eating salted food, fried food or moldy food at least once per week (1 score), family history of upper gastrointestinal cancer in the immediate family and relatives within 3 generations (2 score), having any of these upper gastrointestinal symptoms (2 score), personal history of gastritis, esophagitis or esophageal reflux (2 score). | Score ≥2 | Esophageal or gastric cancer | NA | Rural and urban China |
| Chen WQ, 2020 [68]; Chen WQ 2021 [69] | smoking at least 20 cigarettes per day for last 10 years or more; or smoking tobacco leaf for 10 years or more (1 score); drinking beer at least 5 L per week for last 10 years or more; or drinking white spirit at least 1 L per week and for last 10 years or more (1 score); eating salted food at least once per week (1 score); eating high-temperature food at least once per week (1 score); eating moldy food at least once per week (1 score); family history of digestive system cancer (2 score); Any current symptom of dysphagia, odynophagia, chest pain, back pain, or neck pain (2 score);Any disease history of esophageal reflux or peptic or duodenal ulcer (2 score) | Score ≥2 | Esophageal cancer | Ability to estimate individual risk within 3 years:  Sensitivity: 64.43%;  Specificity: 64.90%;  AUC: 65% | Rural China |

Abbreviations: AUC, area under the curve-receiver operating characteristic; EC, esophageal cancer, NA, not available.

Table S6: Quality assessment of 31 studies reporting compliance with endoscopy examinations included in the meta-analysis

| Study | Item No. | | | | | | | | | | Total Score | Risk of bias |
| --- | --- | --- | --- | --- | --- | --- | --- | --- | --- | --- | --- | --- |
|  | 1 | 2 | 3 | 4 | 5 | 6 | 7 | 8 | 9 | 10 |  |  |
| Lu XJ; 2003 [2] | 0 | 1 | 1 | 0 | 1 | 1 | 1 | 1 | 1 | 1 | 8 | Low |
| Wen DG; 2009 [5] | 0 | 1 | 1 | 0 | 1 | 1 | 1 | 1 | 1 | 1 | 8 | Low |
| Zhang YZ; 2012 [8] | 0 | 1 | 1 | 0 | 1 | 1 | 1 | 1 | 1 | 1 | 8 | Low |
| Wang ZQ; 2014 [10] | 0 | 1 | 1 | 0 | 1 | 1 | 1 | 1 | 1 | 1 | 8 | Low |
| Wei WQ; 2015 [18] | 0 | 1 | 1 | 0 | 1 | 1 | 1 | 1 | 1 | 1 | 8 | Low |
| Wang W; 2016 [22] | 0 | 1 | 1 | 0 | 1 | 1 | 0 | 1 | 1 | 1 | 7 | Moderate |
| Wang M; 2016 [23] | 0 | 1 | 1 | 0 | 1 | 1 | 1 | 1 | 1 | 1 | 8 | Low |
| Jing YH; 2017 [26] | 0 | 1 | 1 | 0 | 1 | 1 | 1 | 1 | 1 | 1 | 8 | Low |
| Xu CL; 2017 [28] | 0 | 1 | 1 | 0 | 1 | 1 | 0 | 1 | 1 | 1 | 7 | Moderate |
| Xu H; 2017 [29] | 0 | 1 | 1 | 0 | 1 | 1 | 0 | 1 | 1 | 1 | 7 | Moderate |
| Zhu JY; 2017 [32] | 0 | 1 | 1 | 0 | 1 | 1 | 0 | 1 | 1 | 1 | 7 | Moderate |
| Liu YZ; 2018 [34] | 0 | 1 | 1 | 0 | 1 | 1 | 0 | 1 | 1 | 1 | 7 | Moderate |
| Sun GF; 2018 [35] | 0 | 1 | 1 | 0 | 1 | 1 | 0 | 1 | 1 | 1 | 7 | Moderate |
| Lin YP; 2019 [40] | 0 | 1 | 1 | 0 | 1 | 1 | 0 | 1 | 1 | 1 | 7 | Moderate |
| Xiao HF; 2019 [43] | 0 | 1 | 1 | 0 | 1 | 1 | 0 | 1 | 1 | 1 | 7 | Moderate |
| Zhang ZY; 2019 [44] | 0 | 1 | 1 | 0 | 1 | 1 | 1 | 1 | 1 | 1 | 8 | Low |
| Guo LW; 2019 [45] | 0 | 1 | 1 | 0 | 1 | 1 | 0 | 1 | 1 | 1 | 7 | Moderate |
| Liu MF; 2019 [47] | 0 | 1 | 1 | 0 | 1 | 1 | 1 | 1 | 1 | 1 | 8 | Low |
| Liu YY; 2020 [50] | 0 | 1 | 1 | 0 | 1 | 1 | 0 | 1 | 1 | 1 | 7 | Moderate |
| Shi J, 2020 [51] | 0 | 1 | 1 | 0 | 1 | 1 | 0 | 1 | 1 | 1 | 7 | Moderate |
| Zhao RC; 2020 [53] | 0 | 1 | 1 | 0 | 1 | 1 | 0 | 1 | 1 | 1 | 7 | Moderate |
| Zhao T; 2020 [54] | 0 | 1 | 1 | 0 | 1 | 1 | 1 | 1 | 1 | 1 | 8 | Low |
| Zhu ZH; 2020 [55] | 0 | 1 | 1 | 0 | 1 | 1 | 0 | 1 | 1 | 1 | 7 | Moderate |
| Zeng HM; 2020 [57] | 0 | 1 | 1 | 0 | 1 | 1 | 0 | 1 | 1 | 1 | 7 | Moderate |
| Lie RJ; 2020 [59] | 0 | 1 | 1 | 0 | 1 | 1 | 0 | 1 | 1 | 1 | 7 | Moderate |
| Li J; 2021 [60] | 0 | 1 | 1 | 0 | 1 | 1 | 1 | 1 | 1 | 1 | 8 | Low |
| Tuo JY; 2021 [61] | 0 | 1 | 1 | 0 | 1 | 1 | 0 | 1 | 1 | 1 | 7 | Moderate |
| Xiong WY; 2021 [62] | 0 | 1 | 1 | 0 | 1 | 1 | 0 | 1 | 1 | 1 | 7 | Moderate |
| Zhang YZ; 2021 [64] | 0 | 1 | 1 | 0 | 1 | 1 | 0 | 1 | 1 | 1 | 7 | Moderate |
| Chen R; 2021 [65] | 0 | 1 | 1 | 0 | 1 | 1 | 1 | 1 | 1 | 1 | 8 | Low |
| Li J; 2021 [66] | 0 | 1 | 1 | 0 | 1 | 1 | 0 | 1 | 1 | 1 | 7 | Moderate |

Table S7: Quality assessment of 44 studies reporting endoscopy findings included in the meta-analysis

| Study | Items | | | | | | | | | | | Total | Risk of bias | |
| --- | --- | --- | --- | --- | --- | --- | --- | --- | --- | --- | --- | --- | --- | --- |
|  | 1 | 2 | 3 | 4 | 5 | 6 | 7 | 8 | 9 | 10 | Score | |  |  |
| Lu XJ; 2003 [2] | 0 | 1 | 1 | 0 | 1 | 1 | 1 | 1 | 1 | 1 | 8 | | Low |  |
| Wang XZ; 2009 [3] | 0 | 1 | 1 | 0 | 1 | 1 | 1 | 1 | 1 | 1 | 8 | | Low |  |
| Yin C; 2009 [4] | 0 | 1 | 1 | 0 | 1 | 0 | 1 | 1 | 1 | 1 | 7 | | Moderate |  |
| Chen LL; 2011 [6] | 0 | 1 | 1 | 0 | 1 | 1 | 1 | 1 | 1 | 1 | 8 | | Low |  |
| Zou WD; 2011 [7] | 0 | 1 | 1 | 0 | 1 | 1 | 1 | 1 | 1 | 1 | 8 | | Low |  |
| Zhang YZ; 2012 [8] | 0 | 1 | 1 | 0 | 1 | 1 | 1 | 1 | 1 | 1 | 8 | | Low |  |
| Guo S; 2013 [9] | 0 | 0 | 0 | 0 | 1 | 1 | 1 | 1 | 1 | 1 | 6 | | Moderate |  |
| Wang ZQ; 2014 [10] | 0 | 1 | 1 | 1 | 1 | 1 | 1 | 1 | 1 | 1 | 9 | | Low |  |
| Yang L; 2014 [11] | 0 | 1 | 1 | 0 | 1 | 1 | 1 | 1 | 1 | 1 | 8 | | Low |  |
| Zhang ZY; 2014 [12] | 0 | 1 | 1 | 0 | 1 | 1 | 1 | 1 | 1 | 1 | 8 | | Low |  |
| Du LB; 2015 [13] | 0 | 1 | 1 | 0 | 1 | 1 | 1 | 1 | 1 | 1 | 8 | | Low |  |
| Liang SY; 2015 [14] | 0 | 1 | 1 | 0 | 1 | 1 | 1 | 1 | 1 | 1 | 8 | | Low |  |
| Lv CH; 2015 [15] | 0 | 1 | 1 | 0 | 1 | 0 | 1 | 1 | 1 | 1 | 7 | | Moderate |  |
| Tuo JY; 2015 [16] | 0 | 1 | 1 | 0 | 1 | 1 | 1 | 1 | 1 | 1 | 8 | | Low |  |
| Zhang M; 2015 [17] | 0 | 1 | 1 | 0 | 1 | 1 | 1 | 1 | 1 | 1 | 8 | | Low |  |
| Wei WQ; 2015 [18] | 0 | 1 | 1 | 0 | 1 | 1 | 1 | 1 | 1 | 1 | 8 | | Low |  |
| Zheng XZ; 2015 [19] | 0 | 0 | 0 | 0 | 1 | 1 | 1 | 1 | 1 | 1 | 6 | | Moderate |  |
| Li ZH; 2016 [20] | 0 | 1 | 1 | 0 | 1 | 1 | 1 | 1 | 1 | 1 | 8 | | Low |  |
| Qian F; 2016 [21] | 0 | 1 | 1 | 0 | 1 | 1 | 1 | 1 | 1 | 1 | 8 | | Low |  |
| Wang M; 2016 [23] | 0 | 1 | 1 | 0 | 1 | 1 | 1 | 1 | 1 | 1 | 8 | | Low |  |
| He X; 2017 [24] | 0 | 1 | 1 | 0 | 1 | 1 | 1 | 1 | 1 | 1 | 8 | | Low |  |
| He YL; 2017 [25] | 0 | 1 | 1 | 0 | 1 | 1 | 1 | 1 | 1 | 1 | 8 | | Low |  |
| Wang X; 2017 [27] | 0 | 1 | 1 | 0 | 1 | 1 | 1 | 1 | 1 | 1 | 8 | | Low |  |
| Zhang N; 2017 [30] | 0 | 1 | 1 | 0 | 1 | 1 | 1 | 1 | 1 | 1 | 8 | | Low |  |
| Zhou TH; 2017 [31] | 0 | 1 | 1 | 0 | 1 | 1 | 1 | 1 | 1 | 1 | 8 | | Low |  |
| Ding GH; 2018 [33] | 0 | 1 | 1 | 0 | 1 | 1 | 1 | 1 | 1 | 1 | 8 | | Low |  |
| Tang X; 2018 [36] | 0 | 1 | 1 | 0 | 1 | 1 | 1 | 1 | 1 | 1 | 8 | | Low |  |
| Cao XQ; 2019 [37] | 0 | 1 | 1 | 0 | 1 | 1 | 1 | 1 | 1 | 1 | 8 | | Low |  |
| Huang YL; 2019 [38] | 0 | 1 | 1 | 0 | 1 | 1 | 1 | 1 | 1 | 1 | 8 | | Low |  |
| Liang F; 2019 [39] | 0 | 0 | 0 | 0 | 1 | 1 | 1 | 1 | 1 | 1 | 6 | | Moderate |  |
| Lin YP; 2019 [40] | 0 | 1 | 1 | 0 | 1 | 1 | 1 | 1 | 1 | 1 | 8 | | Low |  |
| Ma HM; 2019 [41] | 0 | 1 | 1 | 0 | 1 | 1 | 1 | 1 | 1 | 1 | 8 | | Low |  |
| Wang WP; 2019 [42] | 0 | 1 | 1 | 0 | 1 | 1 | 1 | 1 | 1 | 1 | 8 | | Low |  |
| He ZH; 2019 [46] | 0 | 1 | 1 | 0 | 1 | 1 | 1 | 1 | 1 | 1 | 8 | | Low |  |
| Feng X; 2020 [48] | 0 | 1 | 1 | 0 | 1 | 1 | 1 | 1 | 1 | 1 | 8 | | Low |  |
| Jia SC; 2020 [49] | 0 | 1 | 1 | 0 | 1 | 1 | 1 | 1 | 1 | 1 | 8 | | Low |  |
| Shi J; 2020 [51] | 0 | 1 | 1 | 0 | 1 | 1 | 1 | 1 | 1 | 1 | 8 | | Low |  |
| Sun Q; 2020 [52] | 0 | 1 | 1 | 0 | 1 | 1 | 1 | 1 | 1 | 1 | 8 | | Low |  |
| Zhuang Y; 2020 [56] | 0 | 1 | 1 | 0 | 1 | 1 | 1 | 1 | 1 | 1 | 8 | | Low |  |
| Zeng HM; 2020 [57] | 0 | 1 | 1 | 0 | 1 | 1 | 1 | 1 | 1 | 1 | 8 | | Low |  |
| Cao XQ; 2021 [58] | 0 | 1 | 1 | 0 | 1 | 1 | 1 | 1 | 1 | 1 | 8 | | Low |  |
| Zhang J; 2021 [63] | 0 | 1 | 1 | 0 | 1 | 1 | 1 | 1 | 1 | 1 | 8 | | Low |  |
| Chen R; 2021 [65] | 0 | 1 | 1 | 0 | 1 | 1 | 1 | 1 | 1 | 1 | 8 | | Low |  |
| Zhu L; 2022 [67] | 0 | 1 | 1 | 0 | 1 | 1 | 1 | 1 | 1 | 1 | 8 | | Low |  |

Table S8: Sensitivity analysis in publication year, sample size and the number of study centers

| Characteristics | **High-risk areas of EC** | | | |  | **Rural China** | | | |  | **Urban China** | | | |
| --- | --- | --- | --- | --- | --- | --- | --- | --- | --- | --- | --- | --- | --- | --- |
|  | No. of studies | No. of study participants | Pooled rate, %  (95% CI) | *I*^2^ |  | No. of studies | No. of study participants | Pooled rate, %  (95% CI) | *I*^2^ |  | No. of studies | No. of study participants | Pooled rate, %  (95% CI) | *I*^2^ |
| **Publication year** |  |  |  |  |  |  |  |  |  |  |  |  |  |  |
| 1999-2009 | 3 | 16,622 | 2.10 (0.84-5.13) | 99% |  | 0 | NA | NA | NA |  | 0 | NA | NA | NA |
| 2010-2015 | 14 | 205,392 | 1.02 (0.69-1.51) | 98% |  | 0 | NA | NA | NA |  | 0 | NA | NA | NA |
| 2016-2021 | 19 | 654,156 | 0.93 (0.72-1.21) | 97% |  | 4 | 102,413 | 0.48 (0.25-0.93) | 98% |  | 6 | 18,421 | 0.12 (0.07-0.21) | 46% |
| **Sample size** |  |  |  |  |  |  |  |  |  |  |  |  |  |  |
| <5000 | 8 | 19,639 | 2.71 (2.49-2.95) | 97% |  | 0 | NA | NA | NA |  | 4 | 6936 | 0.20 (0.12-0.34) | 0% |
| 5000-9999 | 11 | 77,600 | 0.95 (0.88-1.02) | 94% |  | 1 | 102,413 | 0.18 (0.10-0.33) | 98% |  | 2 | 11,485 | 0.06 (0.03-0.13) | 0% |
| 10000-49999 | 12 | 247,706 | 0.88 (0.61-1.25) | 98% |  | 3 | 96,451 | 0.73 (0.68-0.78) | 99% |  | 0 | NA | NA | NA |
| ≥50000 | 5 | 531,225 | 1.16 (1.13-1.19) | 95% |  | 0 | NA | NA | NA |  | 0 | NA | NA | NA |
| **No. of study center** |  |  |  |  |  |  |  |  |  |  |  |  |  |  |
| Single center | 20 | 183,731 | 1.11 (0.78-1.58) | 98% |  | 0 | NA | NA | NA |  | 3 | 8857 | 0.13 (0.05-0.35) | 70% |
| Multicenter | 16 | 692,439 | 0.95 (0.72-1.24) | 97% |  | 4 | 102,413 | 0.48 (0.25-0.93) | 98% |  | 3 | 9564 | 0.12 (0.06-0.21 | 12% |

Abbreviations: CI, confidence interval; EC, esophageal cancer; NA, not available.


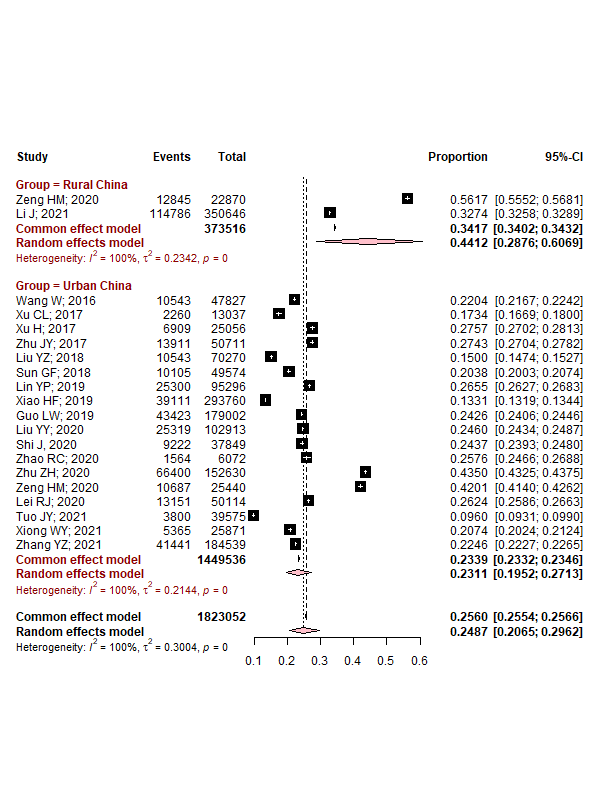
Figure S1: The estimated positivity rate of high-risk individuals in population-based esophageal cancer screening in China, by regions (rural China and urban China)


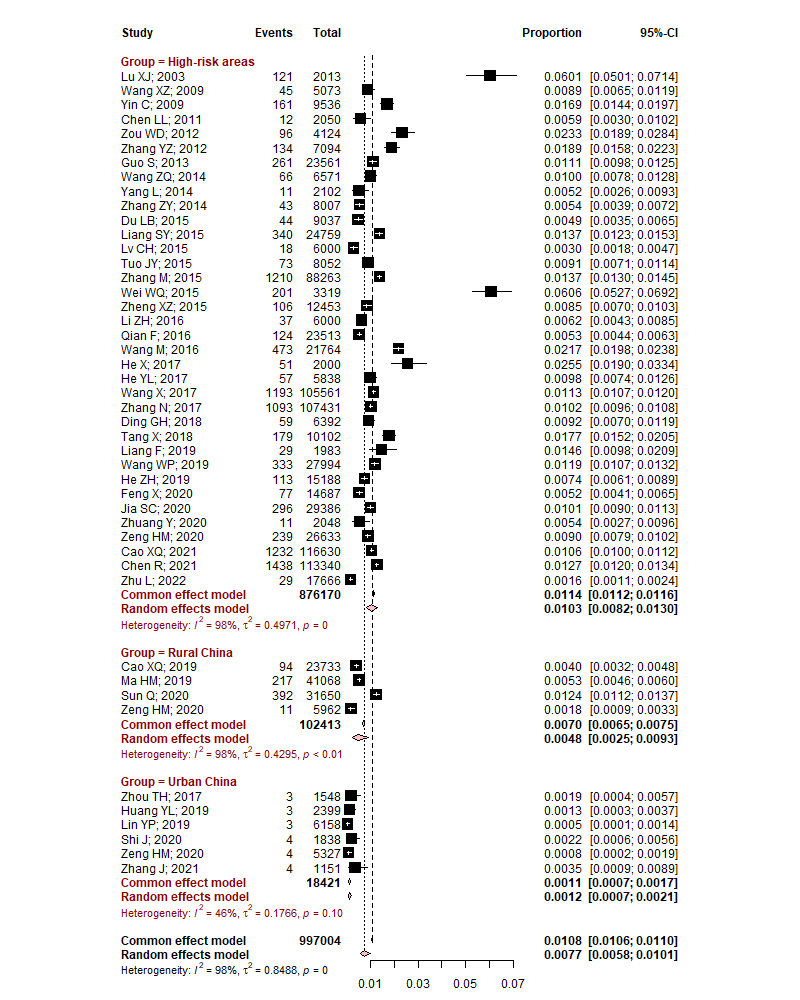
 Figure S2: Estimated detection rates of positive cases in population-based esophageal cancer screening in China, by regions (high-risk areas, rural China and urban China)


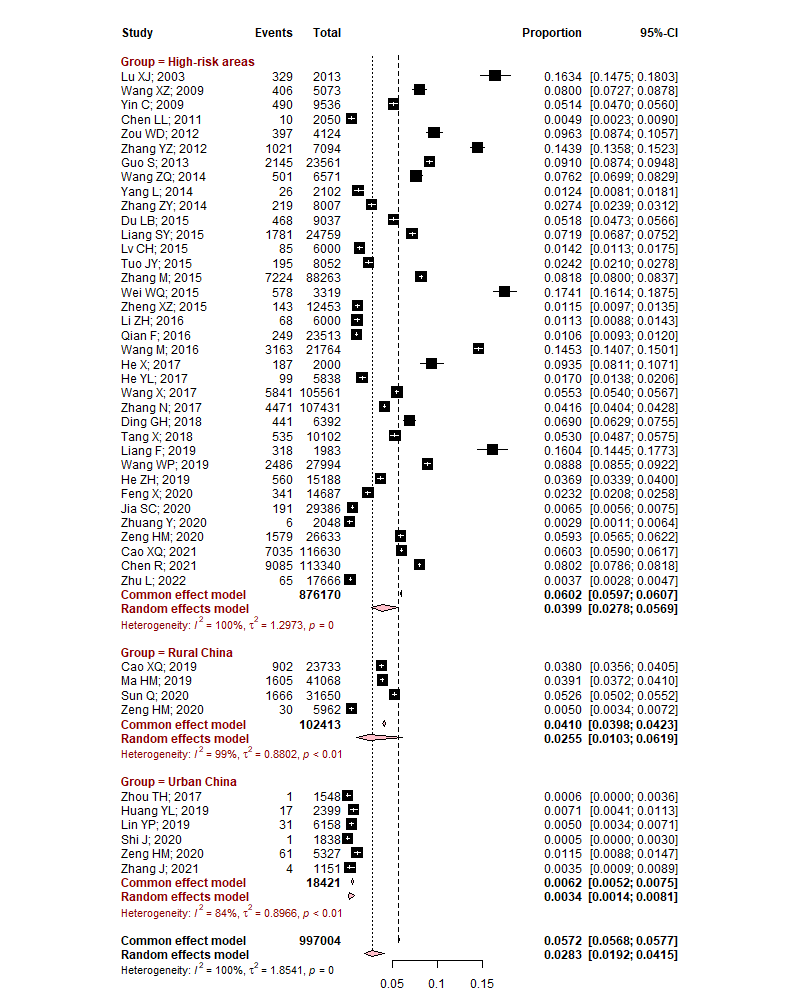


Figure S3: Estimated detection rates of low-grade intraepithelial neoplasia in population-based esophageal cancer screening in China, by regions (high-risk areas, rural China and urban China)


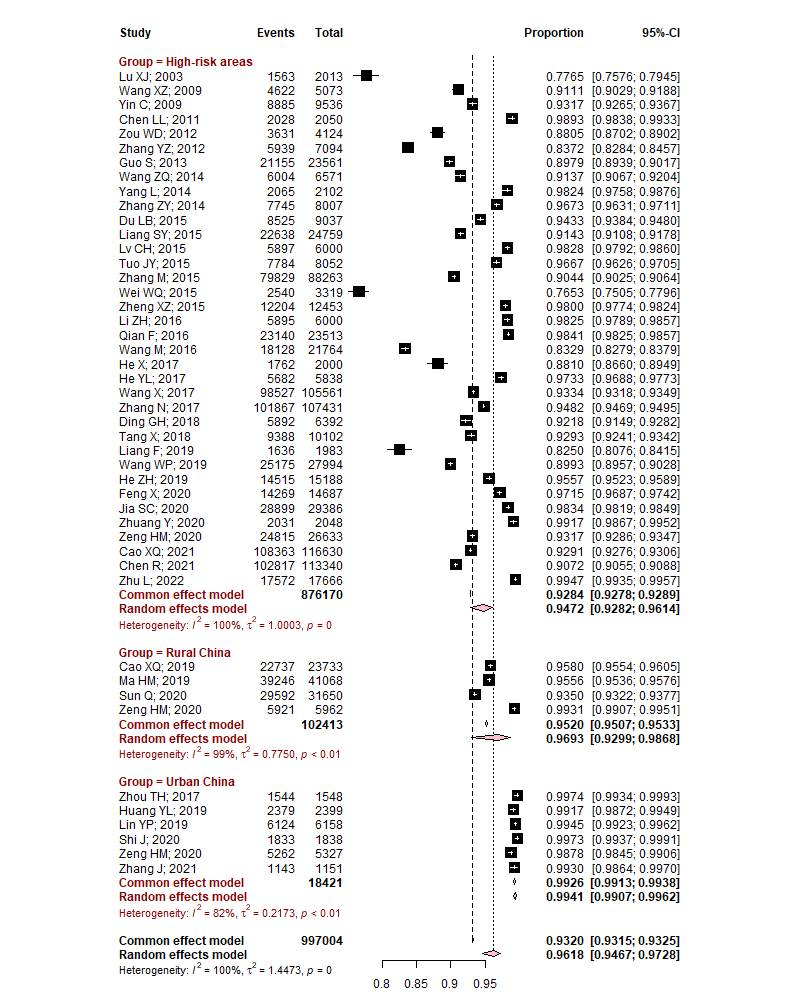


Figure S4: The estimated detection rates of negative endoscopy findings in population-based esophageal cancer screening in China, by regions (high-risk areas, rural China and urban China)


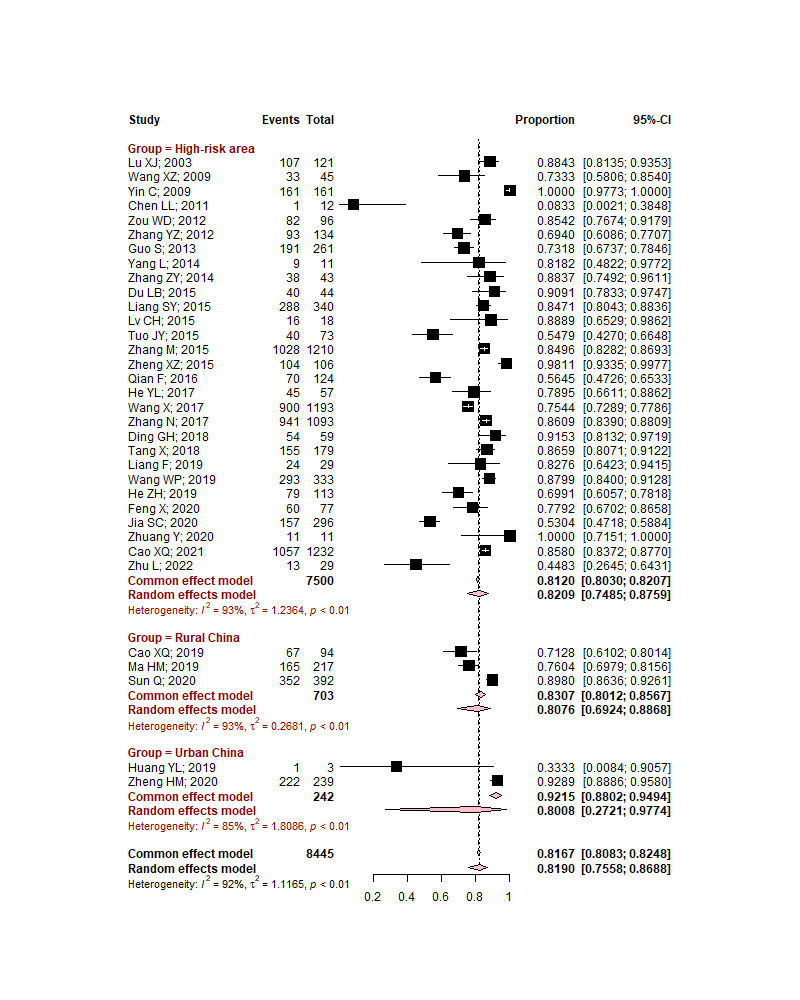


Figure S5: Estimated early detection rates among positive cases in population-based esophageal cancer screening in China, by regions (high-risk areas, rural China and urban China)


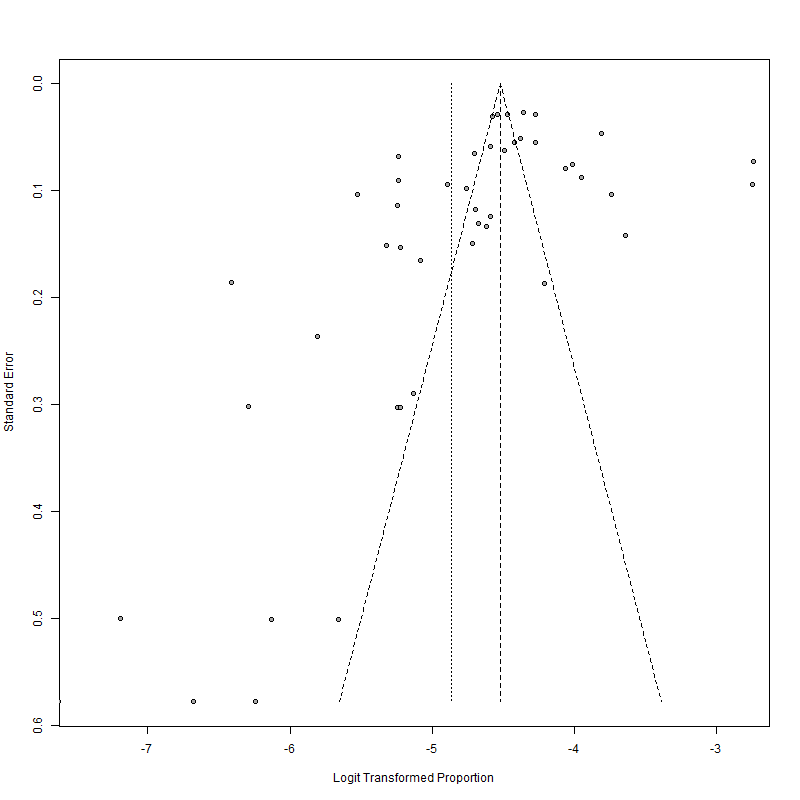


Figure S6: Funnel plot by detection rate of positive cases

**References:**

1. Hoy D, Brooks P, Woolf A, Blyth F, March L, Bain C, et al. Assessing risk of bias in prevalence studies: modification of an existing tool and evidence of interrater agreement. J Clin Epidemiol 2012 Sep;65(9):934-939. [doi: 10.1016/j.jclinepi.2011.11.014]
2. Lu XJ, Hou J, Chen ZF, Guo CL, Li SS, Bai WL, et al. Endoscopic Screening of Esophageal Cancer in High Risk Areas. Article in Chinese. China J Cancer Prev Treat 2003; 10(9): 900-903. [doi: 10.3969/j.issn.1673-5269.2003.09.002]
3. Wang XZ, Zhang YZ, Yuan FM, Wang F, Liu KR. Analysis on 5073 persons of upper gastrointestinal endoscopic screening in Yangcheng county Shanxi province. Article in Chinese. Cancer Research and Clin 2009; 21(9): 641-642+648. [doi: 10.3760/cma.j.issn.1006-9801.2009.09.028]
4. Yin C. The assessment of efficacy of endoscopic staining examination with iodine solution voluntary in high incidence areas of esophageal cancer in Feicheng [dissertation]. Article in Chinese. Shandong Academy of Medical Sciences. 2009. URL: https://d.wanfangdata.com.cn/thesis/ChJUaGVzaXNOZXdTMjAyMzAxMTISCFkxNTc1NTc4GghybHNwajZycQ%3D%3D [accessed 2022-5-31]
5. Wen DG, Wang SJ, Zhang LW, Zhou W, Yu WF, Wang XL. Natural history of esophageal and gastric cardia precursor by repetitive endoscope screening with 425 adults in a high-risk area in China. Cancer epidemiology 2009 Aug; 33(2): 108-112. [doi: 10.1016/j.canep.2009.06.002]
6. Chen LL, Zhang SQ, Yang LN, Liu YQ. Preliminary Report of Screening and Early Detection for Esophageal Cancer and Cardiac Cancer in Gansu Province. Article in Chinese. China Cancer 2011; 20(11): 798-800. URL: https://d.wanfangdata.com.cn/periodical/ChlQZXJpb2RpY2FsQ0hJTmV3UzIwMjMwMTEyEg16Z3psMjAxMTExMDAzGgg1cjdydm1qcw%3D%3D [accessed 2022-5-31]
7. Zou WD. Study on esophageal and cardiac precancerous by endoscopic screening in high-risk area [dissertation]. Article in Chinese. Hebei Medical University. 2011. URL: https://d.wanfangdata.com.cn/thesis/ChJUaGVzaXNOZXdTMjAyMzAxMTISCFkxOTAwMzk5GghtaHVvdmV2dw%3D%3D [accessed 2022-5-31].
8. Zhang YZ, Wang XZ, Zhang M, Ma ZH, Cao L, Shi YT, et al. A Five-year Results Analysis of Esophageal Cancer/Cardia Cancer Screening with Iodine Staining Method through Endoscopy in High-incidence Area of Esophageal Cancer. Article in Chinese. China Cancer 2012; 21(1): 32-34. URL: https://d.wanfangdata.com.cn/periodical/ChlQZXJpb2RpY2FsQ0hJTmV3UzIwMjMwMTEyEg16Z3psMjAxMjAxMDA3GghsY3FqNXU3Nw%3D%3D [accessed 2022-5-31]
9. Guo S. Endoscopic screening and characteristics of the synchronous multiple primary early esophageal and gastric cardia carcinomas and precancerous lesions in high risk areas [dissertation]. Article in Chinese. Hebei Medical University. 2013. URL: https://d.wanfangdata.com.cn/thesis/ChJUaGVzaXNOZXdTMjAyMzAxMTISCFkyMzM3MzAzGgg0Mjc2eWs2Zw%3D%3D [accessed 2022-5-31]
10. Wang ZQ, Zhu D, Tan RR, Song Y, Zhang YJ, Yang F, et al. Analysis of the Screening Results of Esophageal Cancer in Bazhong, Sichuan from 2010 to 2013. Article in Chinese. J Cancer Control Treat 2014; 27(2): 81-84. URL: https://chkdx.cnki.net/kcms/detail/detail.aspx?QueryID=21&CurRec=1&dbcode=CHKJ&dbname=CHKJ1214&filename=SCZF201402006&urlid=&yx=&v=MTA3NDRhTEc0SDlYTXJZOUZZb1I4ZVgxTHV4WVM3RGgxVDNxVHJXTTFGckNVUjdtZlp1Um9GaURuVjdyS05pN1I= [accessed 2022-5-31]
11. Yang L, Ma LT, Yu SX. Investigation and analysis of early diagnosis and treatment project of upper gastrointestinal cancer in Tang County, Hebei Province. Article in Chinese. Ke Ji Feng 2014; (18): 176. [doi: 10.3969/j.issn.1671-7341.2014.18.156]
12. Zhang ZY, Wu ZQ, Lu LZ, Fan P, Zhao GY, Liu JD, et al. Analysis on Screening for Upper Gastrointestinal Cancer in Liangzhou District, Wuwei City, Gansu Province, 2009-2012. Article in Chinese. China Cancer 2014; 23(9): 743-747. [doi: 10.11735/j.issn.1004-0242.2014.09.A008]
13. Du LB, Wang M, Wu W, Li YY, Xu SJ, Mao WM. Analysis of screening results of early diagnosis and treatment of upper gastrointestinal cancer in rural areas of Zhejiang Province. Article in Chinese. Zhejiang Clinical Medical Journal 2015; 17(1): 143-144. URL: https://d.wanfangdata.com.cn/periodical/ChlQZXJpb2RpY2FsQ0hJTmV3UzIwMjMwMTEyEg96amxjeXgyMDE1MDEwODEaCGRmNnVrZjcz [accessed 2022-5-31]
14. Liang SY, Li K, Gong JY, Wang JL, Ma HM, Wang GQ. Results of the endoscopic screening program of esophageal and gastric cardia cancers using iodine staining in Feicheng, Shandong Province, from 2006 to 2012. Article in Chinese. Chin J Oncol 2015; (7): 549-553. [doi: 10.3760/cma.j.issn.0253-3766.2015.07.015]
15. Lv CH. Screening results of early esophageal cancer in Wuning County, Jiangxi Province in recent 4 years. Article in Chinese. Contemporary Medicine 2015; 21(26): 157-158. [doi: 10.3969/j.issn.1009-4393.2015.26.108]
16. Tuo JY, Wei SZ, Li GC, Zhang YL, Zhang M, Xu F, et al. An Analysis the Results of Early Detection and Early Treatment of Esophageal/Cardia Cancer (Upper Gastrointestinal Cancer) in Different Regions of Hubei Province. Article in Chinese. China Cancer 2015; 24(8): 653-656. [doi: 10.11735/j.issn.1004-0242.2015.08.A006]
17. Zhang M, Li X, Zhang SK, Chen Q, Wang FR, Zhang YB, et al. Analysis of effect of screening of esophageal cancer in 12 cities and counties of Henan province. Article in Chinese. Chin J Prev Med 2015; 49(10): 879-882. [doi: 10.3760/cma.j.issn.0253-9624.2015.10.008]
18. Wei WQ, Chen ZF, He YT, Feng H, Hou J, Lin DM, et al. Long-Term Follow-Up of a Community Assignment, One-Time Endoscopic Screening Study of Esophageal Cancer in China. J Clin Oncol 2015 Jun 10;33(17):1951-1957. [doi: 10.1200/JCO.2014.58.0423]
19. Zheng XZ, Mao XH, Xu K, Lü LS, Peng XZ, Wang M, et al. Massive Endoscopic Screening for Esophageal and Gastric Cancers in a High-Risk Area of China. PLoS One 2015 Dec 23;10(12):e0145097. [doi: 10.1371/journal.pone.0145097. PMID: 26699332]
20. Li ZH, Guo HJ. Endoscopic screening for early carcinoma of the upper gastrointestinal tract in 6,000 cases of natural population. Article in Chinese. Modern Digestion & Intervention 2016; 21(1): 32-34. [doi: 10.3969/j.issn.1672-2159.2016.01.010]
21. Qian F, Wang HC, Ge XS, Li JP, Liu XL, Jia JH, et al. Analysis of 23513 cases of precancerous lesions of esophagus screened by gastroscopy in Changyuan County, Henan Province. Article in Chinese. Contemporary Medicine 2016; 22(21): 160-161. [doi: 10.3969/j.issn.1009-4393.2016.21.109]
22. Wang W, Li HZ, Zhu C, Sun XH, Zhang MZ, Wang YQ, et al. 2014-2015 cancer risk assessment and screening in urban population of Zhejiang province. Article in Chinese. Zhe Jiang Yi Xue 2016; (22): 1795-1798+1814. URL: https://d.wanfangdata.com.cn/periodical/ChlQZXJpb2RpY2FsQ0hJTmV3UzIwMjMwMTEyEg16anl4MjAxNjIyMDAzGghsYWVvNnFleA%3D%3D [accessed 2022-5-31]
23. Wang M. A Prospective Cohort Study of the Distribution and the Progress Rate of Precancerous Lesions of Esophageal Squamous Cell Carcinoma in High-risk Areas of China [dissertation]. Article in Chinese. Peking Union Medical College. 2016. URL: https://d.wanfangdata.com.cn/thesis/ChJUaGVzaXNOZXdTMjAyMzAxMTISCFkzMDc1MzQ5GghhYmQzMnUxbA%3D%3D [accessed 2022-5-31]
24. He X, Dong L, Zhao QD, Cao L, Tang X, Li S, et al. Analysis of effect of screening of esophageal cancer of 40-69 year old residents in Langzhong city of Sichuan province. Article in Chinese. Yi Yao Qian Yan 2017; 7(35): 362-363. [doi: 10.3969/j.issn.2095-1752.2017.35.322]
25. He YL, Zhang WJ, Liu YQ. Analysis of Endoscopic Screening for Upper Digestive Tract Cancer in Gaotai County, 2011-2015. Article in Chinese. China Cancer 2017; 26(6): 447-451. [doi: 10.11735/j.issn.1004-0242.2017.06.A007]
26. Jing YH, Yao YM, Shuai PH, Hou W, He X, He Q, et al. Analysis of the Esophageal Cancer Screening for a High Risk Population of 14,000 40~69 Years-old Residents in Nanbu County, Sichuan Province. Article in Chinese. J Cancer Control Treat 2017;30 (1): 49-52. [doi: 10.3969/j.issn.1674-0904.2017.01.010]
27. Wang X, Li B, Bao Y, Wang Y, Wang AR, Qiao L. Efficacy of esophageal cancer screening in high risk population: results of 105 561 subjects in Sichuan province. Article in Chinese. Chin J Oncol 2017; 39(1): 67-71. [doi: 10.3760/cma.j.issn.0253-3766.2017.01.013]
28. Xu CL, Zhou SJ, Li ML, Cao H. Evaluation of the screening effect of "two-step screening of upper gastrointestinal cancer" in with low incidence areas. Article in Chinese. Jiangsu J Prev Med 2017;28 (01): 54-55+63. URL: https://chkdx.cnki.net/kcms/detail/detail.aspx?QueryID=29&CurRec=1&dbcode=CHKJ&dbname=CHKJ1519&filename=JSYF201701017&urlid=&yx=&v=MTA1ODBlWDFMdXhZUzdEaDFUM3FUcldNMUZyQ1VSN21mWnVSb0ZpRGhVN3ZLTHo3U2FMRzRIOWJNcm85RVk0Ujg= [accessed 2022-5-31]
29. Xu H, Wang Q. Assessment on cancer risk and analysis on screening results of urban residents in Nantong from 2015 to 2016. Article in Chinese. Modern Preventive Medicine 2017; 44(20): 3724-3726+3752. URL: https://d.wanfangdata.com.cn/periodical/ChlQZXJpb2RpY2FsQ0hJTmV3UzIwMjMwMTEyEg94ZHlmeXgyMDE3MjAwMTkaCDU2aXNoMmZo [accessed 2022-5-31]
30. Zhang N, Ma HM, Sun YW, Xu CL, Gu JH, Gong JY, et al. Analyzing the result of esophageal cancer screening among rural residents in Shandong province, 2013-2016. Article in Chinese. Chin J Cancer Prev Treat 2017; 24(05): 287-290. URL: https://d.wanfangdata.com.cn/periodical/ChlQZXJpb2RpY2FsQ0hJTmV3UzIwMjMwMTEyEg9xbHpsenoyMDE3MDUwMDEaCDFsbTY5amoy [accessed 2022-5-31]
31. Zhou TH, Gu XF, Zhu JY, Zhu L. Endoscopic Screening of Upper Digestive Tract Cancer Among High-risk Population in Urumqi. Article in Chinese. China Cancer 2017; 26(10): 781-785. URL: https://chkdx.cnki.net/kcms/detail/detail.aspx?QueryID=75&CurRec=1&dbcode=CHKJ&dbname=CHKJ1519&filename=ZHLU201710008&urlid=&yx=&v=MTIwODFYMUx1eFlTN0RoMVQzcVRyV00xRnJDVVI3bWZadVJvRmlEaFdyN01QeVhIZTdHNEg5Yk5yNDlGYklSOGU= [accessed 2022-5-31]
32. Zhu JY, Gu XY, Song SM, Yao F, Zhou TH, Zhu L. Analysis of 2015-2016 population screen of high cancer risk urban residents in Urumqi city. Article in Chinese. J Practical Oncology 2017; 32(04): 367-370. [doi: 10.13267/j.cnki.syzlzz.2017.04.019]
33. Ding GH. Screening for early diagnosis and treatment of Upper Digestive tract Cancer in Wuwei City of Gansu Province and its influencing factors [dissertation]. Article in Chinese. Gansu University Of Chinese Medicine. 2018. URL: https://d.wanfangdata.com.cn/thesis/ChJUaGVzaXNOZXdTMjAyMzAxMTISCUQwMTU1ODA1OBoINHRvcDR0eGM%3D [accessed 2022-5-31]
34. Liu YZ, Kong WX, Luo XH, Dong D, Chen HD, Li N, et al. Analysis of early diagnosis and treatment of cancer in urban populations of Xuzhou city from 2014 to 2016. Article in Chinese. Jiangsu J Prev Med 2018; (4): 382-385+388. URL: https://chkdx.cnki.net/kcms/detail/detail.aspx?QueryID=93&CurRec=1&dbcode=CHKJ&dbname=CHKJ1519&filename=JSYF201804007&urlid=&yx=&v=MjYzMTZyV00xRnJDVVI3bWZadVJvRnlua1U3M01MejdTYUxHNEg5bk1xNDlGWTRSOGVYMUx1eFlTN0RoMVQzcVQ= [accessed 2022-5-31]
35. Sun GF, Zhao E, Xie HF, Zhu JY, Wan L. Analysis on the Result of Early Detection and Treatment of Cancer in 2014 Urumqi. Article in Chinese. Chin Primary Health Care 2018; 32(3): 57-60. [doi: 10.3969/j.issn.1001-568X.2018.03.0021]
36. Tang X, Cao L, Long SZ, Bai YJ, He X, Dong L, et al. Results of the Endoscopic Screening Program of Esophageal Using Iodine Staining in Langzhong, Sichuan Province, from 2012 to 2016. Article in Chinese. J Cancer Control Treat 2018; 31(1): 41-45. [doi: 10.3969/j.issn.1674-0904.2018.01.007]
37. Cao XQ, Guo LW, Liu SZ, Li X, Chen Q, Quan PL, et al. Pathological Distribution in Esophageal Cancer Screening Population in Non-High Incidence Areas. Article in Chinese. China Cancer 2019; 28(10): 731-737. [doi: 10.11735/j.issn.1004-0242.2019.10.A003]
38. Huang YL. Preliminary analysis of early diagnosis and treatment of upper gastrointestinal cancer in Guangxi and clinical application of ME-NBI in screening [dissertation]: Article in Chinese. Guangxi Medical University. 2019. URL: https://d.wanfangdata.com.cn/thesis/ChJUaGVzaXNOZXdTMjAyMzAxMTISCFkzNTU2MDU1Gghwa2VseDhzbQ%3D%3D [accessed 2022-5-31]
39. Liang F, Qiao X, Wang CC, Han CY, Yang BY, Yang SY, et al. Endoscopic iodine staining for clinical investigation of esophageal disease screening in Huai'an of Jiangsu Province with a survey of 1983 cases. Article in Chinese. Chin J Digest Med Imageol (Electronic Edition) 2019; 9(1): 17-21. [doi: 10.3877/cma.j.issn.2095-2015.2019.01.005]
40. Lin YP, Ma J, Zhang Q, Lu YN, Zhang LJ, Zhang X, et al. Analysis of Upper Gastrointestinal Cancer Screening Results in Kunming from 2015 to 2018. Article in Chinese. China Cancer 2019; 28(6): 411-416. [doi: 10.11735/j.issn.1004-0242.2019.06.A003]
41. Ma HM, Shi SD, Chen WQ, Lu PP, Li B, Xu YL, et al. Esophageal Cancer Screening in Five Counties of Southwest Shandong Province from 2007 to 2015. Article in Chinese. China Cancer 2019; 28(10): 738-742. [doi: 10.11735/j.issn.1004-0242.2019.10.A004]
42. Wang WP, Chen SY, Chen JL, Wei JQ, Li AF, Li XM, et al. Screening Results of Upper Gastrointestinal Cancer in 27,994 Normal People. Article in Chinese. J Canc Control Treat 2019; 32(11): 988-992 [doi:10.3969/j.issn.1674-0904.2019.11.007]
43. Xiao HF, Yan SP, Xu KK, Zou YH, Shi ZH, Zhu SL, et al. Analysis of Cancer Screening Program in Changsha Urban Area from 2012 to 2018. Article in Chinese. China Cancer 2019; 28(11): 807-815. [doi: 10.11735/j.issn.1004-0242.2019.11.A001]
44. Zhang ZY, Wu ZQ, Lu LZ, Fan P, Zhao GY, Liu JL, et al. Analysis of the upper gastrointestinal cancer screening and follow-up results in Liangzhou District of Wuwei City from 2009 to 2017. Article in Chinese. Chin J Cancer Prev Treat 2019; 26(23): 1750-1755. [doi: 10.16073/j.cnki.cjcpt.2019.23.02]
45. Guo LW, Zhang SK, Liu SZ, Zheng LY, Chen Q, Cao XQ, et al. Determinants of participation and detection rate of upper gastrointestinal cancer from population-based screening program in China. Cancer Med 2019 Nov;8(16):7098-7107. [doi: 10.1002/cam4.2578]
46. He ZH, Liu Z, Liu MF, Guo CH, Xu RP, Li FL, et al. Efficacy of endoscopic screening for esophageal cancer in China (ESECC): design and preliminary results of a population-based randomised controlled trial. Gut 2019 Feb;68(2):198-206. [doi: 10.1136/gutjnl-2017-315520]
47. Liu MF, He ZH, Guo CH, Xu RP, Li FL, Ning T, et al. Effectiveness of Intensive Endoscopic Screening for Esophageal Cancer in China: A Community-Based Study. Am J Epidemiol 2019 Apr 1;188(4):776-784. [doi: 10.1093/aje/kwy291]
48. Feng X, Hua ZL, Qian DF, Zhou Q, Shi AW, Wei WQ, et al. efficacy of esophageal cancer screening program on population at high risk: a survey carried out in people aged 40-69 years in Yangzhong, Jiangsu province. Article in Chinese. Chin J Epidemiol 2020; 41(6): 908-912. [doi: 10.3760/cma.j.cn112338-20190606-00407]
49. Jia SC, Xu W, Shen XR, Liu R, Zha ZQ, Wang DB, et al. Analysis of esophagus cancer screening results by using idone staining and endoscopy in Anhui Province, China. Article in Chinese. Chin J Cancer Prev Treat 2020; 27(3): 165-171. [doi: 10.16073/j.cnki.cjcpt.2020.03.01]
50. Liu YY, Zuo TT, Yu LZ, Wu Y, Dong SL, Luo YH, et al. Analysis of the results on cancer risk assessment and screening of urban residents in Shenyang. Article in Chinese. Chin J Public Health 2020; 36(1): 1-4. [doi: 10.11847/zgggws1127286]
51. Shi J, Liang D, Xia CJ, Chen SH, Gao W, Wang J, et al. Analysis of Upper Gastrointestinal Cancer Screening Results in Urban Areas of Hebei Province, 2018-2019. Article in Chinese. China Cancer 2020; 29(6): 419-424. [doi: 10.11735/j.issn.1004-0242.2020.06.A003]
52. Sun Q, Pan EC, Sun ZM, Wen JB, Liao DD, Yang H, et al. Analysis of the endoscopic screening results in high incidence areas of esophageal cancer in Huai'an from 2009 to 2017. Article in Chinese. Chin J Cancer Prev Treat 2020;27 (4): 251-255. [doi: 10.16073/j.cnki.cjcpt.2020.04.01]
53. Zhao RC, Lin ZT, Yu WJ, Lei L, Liu Z, Xu Y, et al. Analysis of the prevalence of five cancer risks among permanent residents aged 40-74 years old in Baoan District,Shenz-hen,2017-2019. Article in Chinese. Practical Oncology Journal 2020; 34(4): 315-320. [doi: 10.11904/j.issn.1002-3070.2020.04.005]
54. Zhao T, Li YY, Chen R, Zhang N, Wang JL, Wei WQ, et al. Long-term effects of endoscopic screening for upper gastrointestinal cancer in Feicheng. Article in Chinese. Chin J Cancer Prev Treat 2020; 27(18): 1470-1475. [doi: 10.16073/j.cnki.cjcpt.2020.18.06]
55. Zhu ZH, Cao J, Yu JH, Li QL, Yang ZY, Ge LY, et al. Assessment of cancer risk and analysis of screening results of cancer screening program in urban Guangxi from 2014 to 2017. Article in Chinese. Chin J of Oncol Prev and Treat. 2020; 12(6): 681-685. [doi: 10.3969/j.issn.1674-5671.2020.06.15]
56. Zhuang Y, Zhu L, Zhao PX, Tang LN, Xu LN. Analysis of screening results of early diagnosis and early Treatment Project for Upper gastrointestinal cancer of rural residents in Guizhou Province in 2018. Article in Chinese. Studies of Trace Elements and Health 2020; 37 (6): 55-56. URL: https://d.wanfangdata.com.cn/periodical/ChlQZXJpb2RpY2FsQ0hJTmV3UzIwMjMwMTEyEhJ3bHlzeWpreWoyMDIwMDYwMjYaCGprZDRidHFn [accessed 2022-5-31]
57. Zeng HM, Sun KX, Cao MM, Zheng RS, Sun XB, Liu SZ, et al. Initial results from a multi-center population-based cluster randomized trial of esophageal and gastric cancer screening in China. BMC Gastroenterol 2020 Nov 24;20(1):398. [doi: 10.1186/s12876-020-01517-3].
58. Cao XQ, Zhang SK, Wang FR, Chen Q, Guo LW, Liu SZ, et al. Analysis of the effects of esophageal cancer screening in Henan rural areas with cancer screening program, 2014-2018. Article in Chinese. Chin J Prev Med 2021; 55(2): 184-188. [doi: 10.3760/cma.j.cn112150-20200320-00398]
59. Lei RJ, Zhang MZ, Wang L, Fang ZH, Wang YQ, Li HZ, et al. Analysis of screening results of urban cancer early diagnosis and treatment program in Quzhou city, Zhejiang province, 2014—2018. Article in Chinese. Chin J of Oncol Prev and Treat 2021; 13(6): 618-623. [doi: 10.3969/j.issn.1674-5671.2021.06.08]
60. Li J, Gu JH, He Y, Yuan ZQ, He QG, Xie SH, et al. Incidence of Upper Gastrointestinal Cancer and Endoscopy Screening Results in Yanting County, Sichuan Province. Article in Chinese. China Cancer 2021;30 (5): 346-351. [doi: 10.11735/j.issn.1004-0242.2021.05.A005]
61. Tuo JY, Zhang Z, He XM, Jiang WZ, Feng JP, Jin X, et al. Analysis of the screening results of the urban cancer early diagnosis and treatment project in Hubei province from 2018 to 2019. Article in Chinese. Practical Oncology Journal 2021; 35(4): 297-301. [doi: 10.11904/j.issn.1002-3070.2021.04.002]
62. Xiong WY, Chen HY, Feng XW, Fan YB, Sun YS, Zhu XL, et al. Assessment and screening results of risk of upper gastrointestinal cancer in urban inhabitants located in Nanchang from 2018 to 2019. Article in Chinese. Chin J Clin Oncol Rehebil 2021; 28(11): 1316-1320. [doi: 10.13455/j.cnki.cjcor.2021.11.10]
63. Zhang J, Wei DH, Yin HP, Ma YL. Analysis of esophageal cancer and precancerous lesions in high-risk groups of esophageal cancer. Article in Chinese. Journal of Taishan Medical College 2021; 42(1): 10-12. [doi: 10.3969/j.issn.1004-7115.2021.01.003]
64. Zhang YZ, Gao QS, Cui WF, Cao L, Guo XR, Ma ZH, et al. Analysis of Cancer Screening Program in Shanxi Urban Area from 2014 to 2018. Article in Chinese. China Cancer 2021; 30(02): 131-136. [doi: 10.11735/j.issn.1004-0242.2021.02.A005]
65. Chen R, Liu Y, Song GH, Li BY, Zhao DL, Hua ZL, et al. Effectiveness of one-time endoscopic screening programme in prevention of upper gastrointestinal cancer in China: a multicentre population-based cohort study. *Gut* 2021 Feb;70(2):251-260. [doi: 10.1136/gutjnl-2019-320200]
66. Li J, Li H, Zeng HM, Zheng RS, Cao MM, Sun DQ, et al. Trends in high-risk rates and screening rates for the population-based cancer screening program on esophageal, stomach and liver cancer in China, 2010-2016. J Natl Cancer Cent 2021 Sep; 1(3): 101-107. [doi: 10.1016/j.jncc.2021.05.001]
67. Zhu L, Hu YD, Zhao FX, Xu LN, Yu ZR, Liu T. Screening of Upper Digestive Tract Cancer in Rural Areas of Guizhou Province, 2009-2019. Article in Chinese. China Cancer 2022; 31(11):903-908. [doi: 10.11735/j.issn.1004-0242.2022.11.A009]
68. Chen WQ, Li H, Zheng RS, Ren JS, Shi JF, Cao MM, et al. An initial screening strategy based on epidemiologic information in esophageal cancer screening: a prospective evaluation in a community-based cancer screening cohort in rural China. *Gastrointest Endosc* 2021 Jan;93(1):110-118.e2. [doi: 10.1016/j.gie.2020.05.052]
69. Chen WQ, Li H, Ren JS, Zheng RS, Shi JF, Li J, et al. Selection of high-risk individuals for esophageal cancer screening: A prediction model of esophageal squamous cell carcinoma based on a multicenter screening cohort in rural China. *Int J Cancer* 2021 Jan 15;148(2):329-339. [doi: 10.1002/ijc.33208]
